# Tissue-specific impacts of aging and genetics on gene expression patterns in humans

**DOI:** 10.1038/s41467-022-33509-0

**Published:** 2022-10-03

**Authors:** Ryo Yamamoto, Ryan Chung, Juan Manuel Vazquez, Huanjie Sheng, Philippa L. Steinberg, Nilah M. Ioannidis, Peter H. Sudmant

**Affiliations:** 1 Department of Integrative Biology, University of California Berkeley, Berkeley, USA; 2 Bioinformatics Interdepartmental Program, University of California Los Angeles, Los Angeles, USA; 3 Center for Computational Biology, University of California Berkeley, Berkeley, USA; 4 Department of Electrical Engineering and Computer Sciences, University of California Berkeley, Berkeley, USA

**Keywords:** Gene expression, Transcriptomics, Evolutionary genetics

## Abstract

Age is the primary risk factor for many common human diseases. Here, we quantify the relative contributions of genetics and aging to gene expression patterns across 27 tissues from 948 humans. We show that the predictive power of expression quantitative trait loci is impacted by age in many tissues. Jointly modelling the contributions of age and genetics to transcript level variation we find expression heritability (*h*^2^) is consistent among tissues while the contribution of aging varies by >20-fold with ${R}_{{{{{{{{\rm{age}}}}}}}}}^{2} \; > \;{h}^{2}$ in 5 tissues. We find that while the force of purifying selection is stronger on genes expressed early versus late in life (Medawar’s hypothesis), several highly proliferative tissues exhibit the opposite pattern. These non-Medawarian tissues exhibit high rates of cancer and age-of-expression-associated somatic mutations. In contrast, genes under genetic control are under relaxed constraint. Together, we demonstrate the distinct roles of aging and genetics on expression phenotypes.

## Abstract (web summary)

Age is a risk factor for many diseases, but the impact of aging on molecular phenotypes is not fully understood. Here, the authors quantify the relative contributions of genetics and aging to gene expression patterns across 27 tissues in humans, showing that age and genetics each play distinct roles in shaping expression phenotypes.

## Introduction

Organismal survival requires molecular processes to be carried out with the utmost precision. However, as individuals age many biological processes deteriorate resulting in impaired function and disease. Such increases in the overall variance of molecular processes are predicted by Medawar’s germline mutation accumulation theory^1^, which states that because older individuals are less likely to contribute their genetic information to the next generation, there is reduced selection to eliminate deleterious phenotypes that appear late in life^2^. This theory also predicts that genes expressed early in life should be under increased selective constraint compared to genes expressed late in life. However, a key challenge remains in both quantifying age-associated changes in biological processes across tissues and identifying how genetic variation influences such changes.

At the organismal level, age-associated changes in the heterogeneity of gene expression between individuals have been observed for a handful of genes in humans^3^. In an analysis of gene expression in monozygotic (identical) twins, 42 genes showed age-associated differences in gene expression, suggesting a role for the environment in modulating gene expression with age^2,3^. Similarly, the number of genes with expression quantitative trait loci (eQTLs) detected from blood in 70 year olds declined by 4.7% when they were resampled at 80 years old^4^. However, the extent of this phenomenon, both across genes and tissues, remains unclear^5^. Age-associated increases in the heterogeneity of gene expression have also been observed at the level of individual cell-to-cell variation; however, only some cell types appear to be impacted^6^. In a recent study of immune T-cells from young and aged individuals, no difference in cell-to-cell variability was observed in unstimulated cells, however, upon immune activation the older cells appeared more heterogeneous^7^. It is not known why some cell-types and not others may be more likely to exhibit increased cellular variability.

The relationship between the age at which a specific gene is expressed and the force of purifying selection has also recently been explored across a number of species^8,9^. These analyses have broadly confirmed that, on average, genes expressed later in life are under less constraint compared to those expressed early in life. However, how these patterns vary across different tissues and are impacted by genetic variation has not been systematically explored.

Here we set out to understand how aging affects the molecular heterogeneity of gene expression and to model the relative impact of age and genetic variation on this phenotype across tissues. First, using gene expression data from 948 individuals in GTEx V8^10^ we show that age impacts the predictive power of eQTLs, however to varying extents across different tissues and in old and young individuals. Increases in between-individual gene expression heterogeneity were associated with these reductions in eQTL power. Using a regularized linear model-based approach to jointly model the impact of both age and genetic variation on gene expression we find that while the average heritability of gene expression is consistent across tissues, the average contribution of age varies substantially. Furthermore, while the genetic regulation of gene expression is similar across tissues, age-associated changes in gene expression are highly tissue-specific in their action. We use this joint model to identify each gene’s age of expression and show that while in most tissues late-expressed genes do tend to be under more relaxed selective constraint, among a handful of highly proliferative tissues the opposite trend holds.

## Results

### Expression quantitative trait loci exhibit varying predictive power in *old* and *young* individuals across several different tissues

**Fig. 1:**
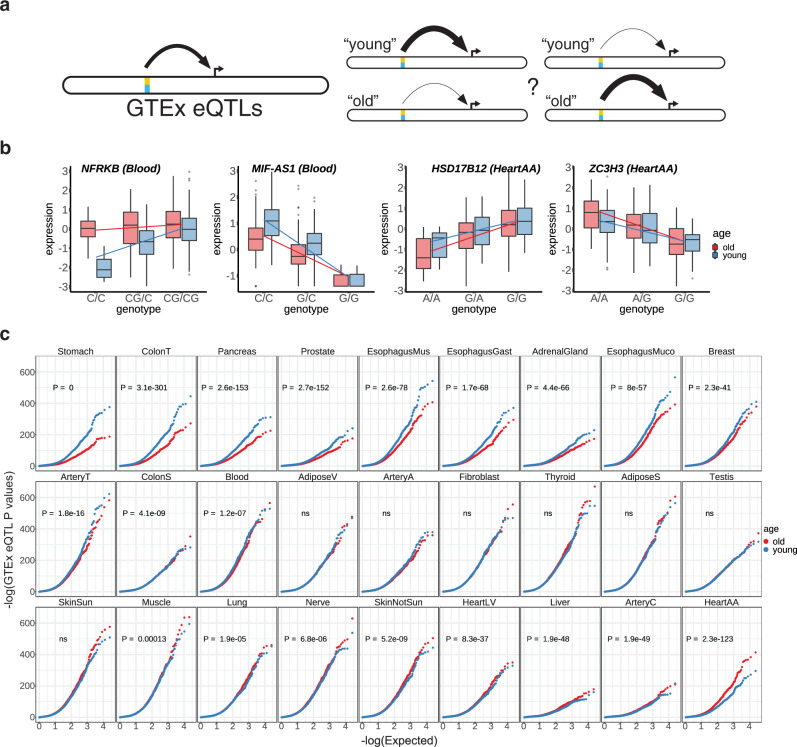
Age impacts the predictive power of eQTLs in many tissues. **a** A hypothetical model of the differing power to detect eQTLs in old and young cohorts. **b** Examples of gene expression binned by genotype and age for four genes in which eQTL *p*-values differ between old and young individuals in whole blood and heart atrial appendage. Example genes with both positive and negative effects are shown. *n* = 309 samples for old group in blood, *n* = 361 samples for young group in blood, *n* = 202 samples for old group in heart atrial appendage, *n* = 170 samples for young group in heart atrial appendage. Center line of the boxplot indicates median, box limit indicates first and third quartiles and points indicate outliers. **c** QQ plots of eQTL p-values (plotted as -log(*P*)) for old (red) and young (blue) individuals from a linear model correlating expression with the lead SNP for each gene in 27 tissues (Supplementary Data 2). P-values for significant differences in eQTL p-value distributions are obtained from a two-sided Welch's *t*-test.

To gain insight into how gene regulatory programs might be impacted by aging, we analyzed transcriptomic data collected across multiple tissues from 948 humans (GTEx version 8)^10^. We hypothesized that aging might dampen the effect of expression quantitative trait loci (eQTLs) due to factors such as increased environmental variance or molecular infidelity (Fig. 1a, b). To test this hypothesis we first classified individuals into old and young age groups, conservatively grouping individuals above and below the median age (55 years old, Supplementary Fig. 1), respectively, and restricting our analyses to tissues with at least 100 individuals in both groups (27 tissues in total, Supplementary Fig. 2, Supplementary Data 1). In each tissue we down-sampled to match the sample size of old and young individuals while additionally controlling for co-factors such as ancestry and technical confounders (Methods). Of note, a common approach to controlling for unobserved confounders in large gene expression experiments is to probabilistically infer hidden factors using statistical tools such as PEER^11^. We noticed that many of the GTEx PEER factors were significantly correlated with sample age, with the top three correlated PEER factors having a Pearson r of 0.33, −0.21, and −0.15 (Supplementary Fig. 3). To prevent loss of age related variation, we recalculated a corrected set of PEER factors that were independent of sample age (Methods). We then assessed the significance of GTEx eQTLs in the young and old cohorts respectively, comparing the distribution of P-values over all genes between old and young individuals (Fig. 1c, two-sided Welch’s *t*-test). In 20 out of 27 (74%) of the assessed tissues, the *P*-value distribution was significantly different between young and old individuals, with genotypes more predictive of expression in younger individuals in 12/20 cases. While 8 tissues show the opposite trend, with increased predictive power in older individuals compared to younger individuals, the magnitude of this effect was significantly reduced (Wilcoxon rank sum test, *P* = 0.031, Supplementary Fig. 4). These results were largely identical when the analyses were performed with the original non-corrected PEER factors (18/27 tissues, Supplementary Fig. 5). This effect was not significantly associated with the number of eGenes identified by GTEx (Supplementary Fig. 6).

While the GTEx dataset is unique in its wide sampling of participant ages and tissues, we validated our observations in the PIVUS cohort which includes blood tissue from individuals re-sampled at ages 70 and 80^4^. This study previously demonstrated a reduction in eQTL heritability with age, supporting our results. We confirmed using our approach that eQTLs were less predictive of gene expression in 80, compared to 70 year olds (Supplementary Figs. 7, 8). These results suggest that the predictive power of eQTLs is impacted by the sample age across the vast majority of tissues. Furthermore, this effect is more pronounced in older samples compared with younger samples.

### Age-associated changes in gene expression heterogeneity impact gene expression heritability

**Fig. 2:**
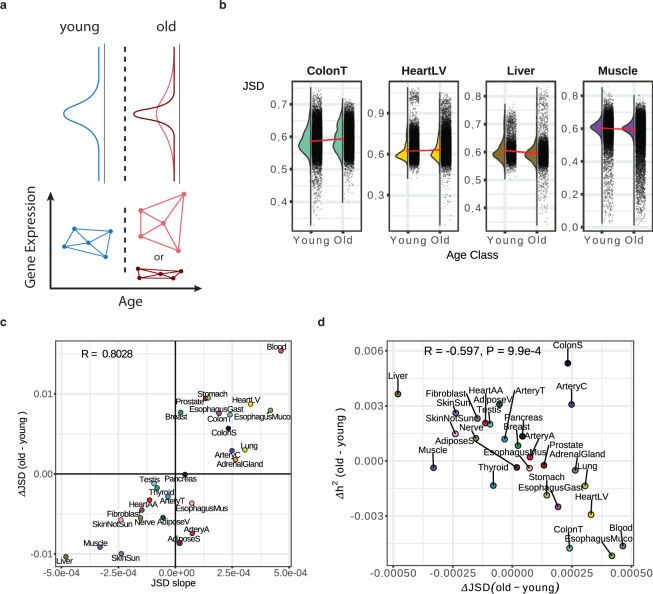
Inter-individual gene expression heterogeneity changes with age for a subset of tissues. **a** Hypothesized age-associated changes in gene expression heterogeneity (top) and our approach for quantifying the inter-individual expression distance with age using the Jensen Shannon Divergence metric (JSD) for age-binned individuals (bottom). **b** The distributions of JSD distances for four example tissues in old and young bins. Red points show the means for each group. Cloud plots show individual pairwise distances and half violin plots show distributions. **c** Consistency of measuring the average age-associated change in gene expression heterogeneity across a tissue using a binary binning strategy (y-axis, *JSD*_*old*_-*JSD*_*young*_) or a 6 bin strategy (x-axis, slope of JSD across 6 age bins). R indicates Pearson correlation value. **d** The relationship between gene expression heterogeneity and the difference in expression heritability between old and young individuals. R and p-value are obtained from two-sided Pearson correlation test.

We hypothesized that the overall reduced predictive power of eQTLs in some tissues might be in part due to an increase in expression heterogeneity in these tissues, potentially as a result of increased environmental variance. To test if such an effect would broadly affect expression across all genes in a tissue (Fig. 2a), we calculated the distribution of pairwise distances among individual’s tissue-specific gene expression profiles using the Jensen-Shannon Divergence (JSD)^12,13^ as a distance metric. The JSD is a robust distance which is less impacted by outliers compared to other methods (e.g., Euclidean distance)^13^. Comparing the distribution of pairwise differences in transcriptional profiles within distinct age groups allows us to determine if gene expression signatures are more similar among younger individuals or among older individuals.

We compared the mean difference in gene expression distances among old and young individuals as well as the slope of the inter-individual JSD when grouping individuals into six bins spanning 20−80 years old (see Methods, Fig. 2b, c). These two strategies yielded highly similar results (Fig. 2c Pearson’s *R* = 0.8) identifying tissues exhibiting increased heterogeneity in both young and old populations. (Supplementary Fig. 9) Thus, contrary to our initial hypothesis, aging does not universally result in increased heterogeneity in gene expression patterns. The difference in JSD between old and young individuals was also negatively correlated with the results from our analysis of eQTLs across old and young individuals (Supplementary Fig. 10, *R* = −0.48, *P* = 0.01 two-sided Pearson correlation test) highlighting that tissues with increases in inter-individual heterogeneity were likely to also exhibit reductions in the proportion of variance explained by eQTLs.

To expand our eQTL analyses to account for the combined impact of nearby SNPs, we utilized the multi-SNP regularized linear model developed in PrediXcan^14^. This model has the benefit of combining genetic effects across many loci, instead of examining just a single eQTL variant. This combined genetic contribution to gene expression variance results in an estimate of the heritability (*h*^2^) for each gene. We applied this model independently in old and young individuals to quantify *h*^2^ and found that the average per-gene difference in *h*^2^ between old and young individuals was strongly negatively correlated with the difference in JSD between samples (Pearson’s *R* = −0.6, *P* = 9.9e-4 Pearson correlation test, Fig. 2d, Supplementary Fig. 11). To verify these results we again referred to the PIVUS study and obtained cis heritability estimates using the GCTA package^15^. As expected, we observed that the heritability of gene expression decreases with age, corresponding with the PrediXcan results in GTEx whole blood (Supplementary Fig. 12). Together, these results suggest that across numerous tissues gene expression heterogeneity differs between young and old individuals. Increased expression variance, either in old or in young individuals, drives a reduction in the average heritability of gene expression across these tissues.

We additionally sought to identify individual genes exhibiting age-associated expression heterogeneity by testing if, after regressing out age-related changes in gene expression levels, the variance of the residuals correlated with age (*Breusch-Pagan test*). The effect size from this test (*β*_*het*_) describes the strength and direction of age related changes in gene expression variance. Using this approach we identified 279 genes with age-associated variance changes (FDR < 0.05) across tissues (Supplementary Fig. 13). The estimated *β*_*het*_ values in these genes were overwhelmingly negative (234/279, 84%, Supplementary Data 3) indicating that the dominant signature was of reduced gene expression heterogeneity with age. A Gene Set Enrichment Analysis (GSEA) of these genes highlighted pathways involved in metabolism, cell proliferation, cell cycle and cell death (Supplementary Fig. 14). While the proportion of positively heteroskedastic genes was weakly correlated with the transcriptome-wide JSD (*P* = 1.32e-2 two-sided Pearson correlation test, Supplementary Fig. 15), the small number of genes implicated suggests that these metrics are capturing different phenomena.

### Cell-type specific age-associated changes in gene expression heterogeneity and the predictive power of eQTLs

While no datasets of the magnitude and scale of GTEx exist for single-cell genomic data, we employed the tool CIBERSORT^16^ to deconvolute bulk GTEx blood RNA-seq data into cell-type specific abundances. Assessing the predictive power of eQTLs in old and young individuals in six immune cell subtypes we found significantly increased explanatory power of eQTLs in younger individuals compared to older individuals (Supplementary Fig. 16). Consistent with these analyses, a comparison of the JSD in old and young individuals revealed increased expression heterogeneity across these cell types with age (Supplementary Fig. 17). We also investigated whether the observed differences in eQTL power and expression heterogeneity might be driven by changes in cell-type composition; however, cell-type composition changes were not reflective of gene expression variance (*P* = 0.2 two-sided Pearson correlation test, Supplementary Fig. 18), suggesting that age associated changes in eQTL powerand expression heterogeneity are taking place at the transcript level.

### Jointly modeling the impact of age and genetics on gene expression identifies distinct, tissue-specific patterns of aging

**Fig. 3:**
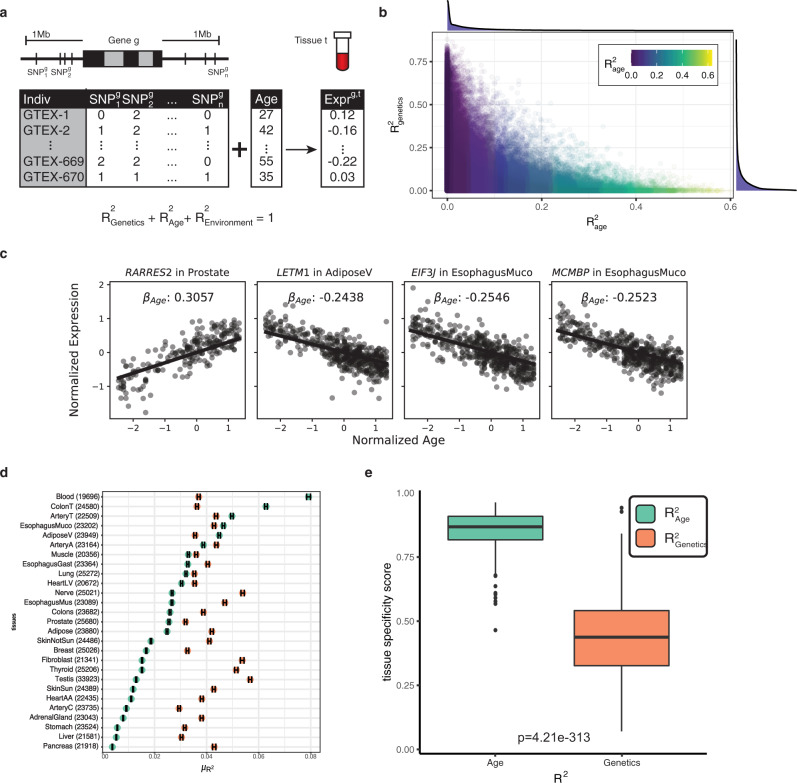
A joint predictive model of gene expression identifies tissue-specific contributions of age and genetics to transcript levels. **a** A schematic of our multi-SNP gene expression association model incorporating sample age. Common SNPs around each gene *g* are used in combination with an individual's age to predict expression within tissue *t*. Using this trained model, variation in gene expression can be separated into three parts: the components explained by genetics (${R}_{{{{{{{{\rm{genetics}}}}}}}}}^{2}$ or *h*^2^), by age (${R}_{{{{{{{{\rm{age}}}}}}}}}^{2}$) and by all other factors (${R}_{{{{{{{{\rm{environment}}}}}}}}}^{2}$). **b** Proportion of each gene's expression variance explained by age and genetics. **c** Plot of normalized expression vs age for four genes with age-correlated expression. Line shows fitted *β*_age_ from regularized linear model. **d** Point estimates of the mean ${R}_{{{{{{{{\rm{age}}}}}}}}}^{2}$ and *h*^2^ for each tissue, error bar indicating standard error for the estimate. **e** The tissue specificity score of *R*^2^ across 27 tissues for each gene from either age or genetics. Center line of the boxplot indicates median, box limit indicates first and third quartiles, points on both ends indicate minima and maxima. *P*-value is obtained from two-sided paired samples *t*-test with *n* = 812 genes.

A more powerful approach to understand how both genetics and age impact gene expression variation is to jointly model these factors simultaneously. We set out to extend the regularized linear model employed by PrediXcan^14^ to incorporate an age factor (Fig. 3a), allowing us to parse apart the individual contributions of genetics (${R}_{{{{{{{{\rm{genetics}}}}}}}}}^{2}$ or *h*^2^), age (${R}_{{{{{{{{\rm{age}}}}}}}}}^{2}$), and the environment (${R}_{{{{{{{{\rm{environment}}}}}}}}}^{2}$) to the expression variance of each gene (e.g., Fig. 3b, c and Supplementary Fig. 19). We define ${R}_{{{{{{{{\rm{environment}}}}}}}}}^{2}$ as all sources of variation not captured by *h*^2^ and ${R}_{{{{{{{{\rm{age}}}}}}}}}^{2}$. Estimates of *h*^2^ in our extended model were highly consistent with those in the original PrediXcan approach (Supplementary Fig. 20).

Employing our model across each tissue independently we find that average heritability of gene expression is largely consistent among tissues, ranging from 2.9% to 5.7%, with 40% of genes having an *h*^2^ > 10% in at least one tissue (Fig. 3d, Supplementary Fig. 21). Thus, while the variation in expression of many individual genes is strongly influenced by genetics, on average, genetics explains a small proportion of overall gene expression variation. In contrast, the average contribution of aging to gene expression varied more than 20-fold among tissues from 0.4% to 7.9%, with the average ${R}_{{{{{{{{\rm{age}}}}}}}}}^{2}$ greater than the average *h*^2^ in 5 tissues. Among these 5 tissues the expression of 39–54% of genes was more influenced by age than by genetics (i.e. ${R}_{{{{{{{{\rm{age}}}}}}}}}^{2}$ > *h*^2^, Supplementary Fig. 22), and across all tissues 45% of genes had an ${R}_{{{{{{{{\rm{age}}}}}}}}}^{2}$ > 10% in at least one tissue. Assessing the tissue-specificity of these trends on a per-gene basis we found that while the estimated heritability of gene expression tended to be similar among different tissues, the age-associated component exhibited significantly more tissue specificity (*P* = 4.21e-313 two-sided paired *t*-test, Fig. 3e). We note that the widespread signatures of age-associated gene expression variance that we identify are virtually undetectable when using the GTEx-provided PEER factors. Just 1.84% of the age-associated genes we identify have nonzero age coefficients when using these GTEx PEER factors (Supplementary Fig. 23). We tested if sex-specific age effects were contributing to the observed age associations, as might be expected if changes related to menopause were playing a role (Supplementary Fig. 24). Including an interaction term between age and sex in our joint model we found that while the age term continued to describe a large proportion of the variance (on average 2.6%), the contribution of the age-sex interaction term was several-fold lower (average of 0.035%, Supplementary Fig. 25, Supplementary Data 4). The model incorporating age-sex interactions also showed consistent estimates of variance explained as compared to the baseline joint model (*R* = 0.99, Supplementary Fig. 26). Our model thus widely expands the utility of the GTEx dataset for exploration of critical biological signatures of aging. Together, these results imply that age-associated patterns of gene expression exhibit substantially more tissue specificity than those that are influenced by genetics and among several tissues age plays a much stronger role in driving gene expression patterns than genetics.

### Coordinated decline of mitochondrial and translation factors is a widespread signature of aging across tissues

**Fig. 4:**
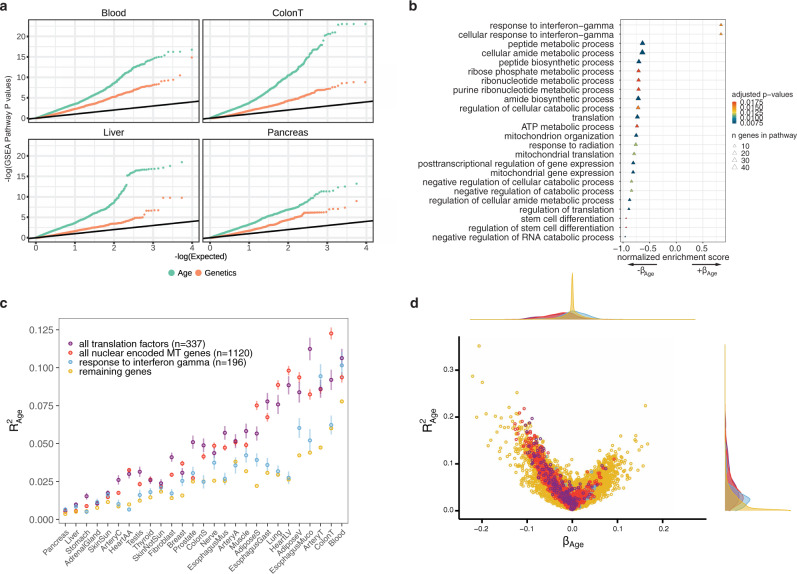
Functional analysis of age-related genes reveals enriched biological processes. **a** A QQ plot of p-values for pathways tested for enrichment using gene set enrichment analysis (GSEA) with genes ranked by either *h*^2^ or ${R}_{{{{{{{{\rm{age}}}}}}}}}^{2}$ in four example tissues. **b** GSEA enrichments from genes ranked by the mean *β*_age_ across tissues. *P*-values were obtained using two-sided permutation test. Pathways with a multiple testing corrected *P* < 0.02 are shown. **c** Average gene expression variance explained by age for mitochondrial (MT) genes (red), translation factor genes (purple), interferon gamma genes (blue) and remaining genes (yellow) across all tissues, error bar indicating standard error of mean. **d** Volcano plot of the variance explained by age vs *β*_age_ for mitochondrial, translation factors, interferon gamma factors, and remaining genes. Density plot of each axis shown on top and right.

To understand the underlying biological implications of age-associated gene expression changes we applied gene set enrichment analysis (GSEA)^17^ to each tissue independently, ranking genes either by the relative contribution of genetics (*h*^2^) or aging (${R}_{{{{{{{{\rm{age}}}}}}}}}^{2}$). Comparing the distribution of P-values from enriched GO-annotations we found that pathways enriched for age-associated variance were substantially more significant than pathways associated with genetic-associated variance (e.g. Fig. 4a). We found more age-associated pathway enrichment even in tissues for which the average age-associated contribution to gene expression was low (e.g. Pancreas, Supplementary Fig. 27). This implies that while age-associated changes in gene expression vary widely in their magnitude among tissues, these changes consistently impact critical biological processes. A GSEA of genes ranked by the tissue-averaged slope of the age-associated trend (*β*_age_) highlighted several key aging-associated pathways^18,19^. Pathways associated with various mitochondrial and metabolic processes and translation were enriched for having − *β*_age_ values, implying age-associated decreases in expression (Fig. 4b). A single immune pathway, the interferon-gamma response, was enriched having + *β*_age_ values. An additional 18 immune pathways were identified as having age-associated increases in gene expression using a more lenient significance threshold (FDR < 0.05) (Supplementary Fig. 28, Supplementary Data 5). In contrast, no pathways were significantly enriched when genes were ranked by average *h*^2^.

To further explore the functional impact of age-associated gene expression changes we compared the ${R}_{{{{{{{{\rm{age}}}}}}}}}^{2}$ of all nuclear-encoded mitochondrial genes^20^ (*n* = 1120), and translation initiation, elongation, and termination factors across tissues (Fig. 4c, Supplementary Fig. 29). Genes in these pathways were exceptionally enriched for age-associated gene expression across several tissues. In some cases >10% of the average expression variation of mitochondrial or translation factor genes could be explained by age. *β*_age_ was consistently negative in these mitochondrial and translation factor genes (Fig. 4d) highlighting that genes in these pathways exhibit a systematic decrease in expression as a function of age. Overall across tissues an average of 36% of all mitochondrial genes (406/1120) and 35% of translation factors (119/337) exhibited age-associated declines, however in some tissues these proportions exceeded 60%. In contrast, the only pathway associated with age-associated increases in expression, interferon-gamma response genes, was largely specific to blood and arterial tissue (Fig. 4c), likely due to the role of this pathway in immune cells. Together these results demonstrate that the coordinated decline of mitochondrial genes and translation factors is a widespread phenomenon of aging across several tissues with potential phenotypic consequences.

### Distinct evolutionary signatures of gene expression patterns influenced by aging and genetics

**Fig. 5:**
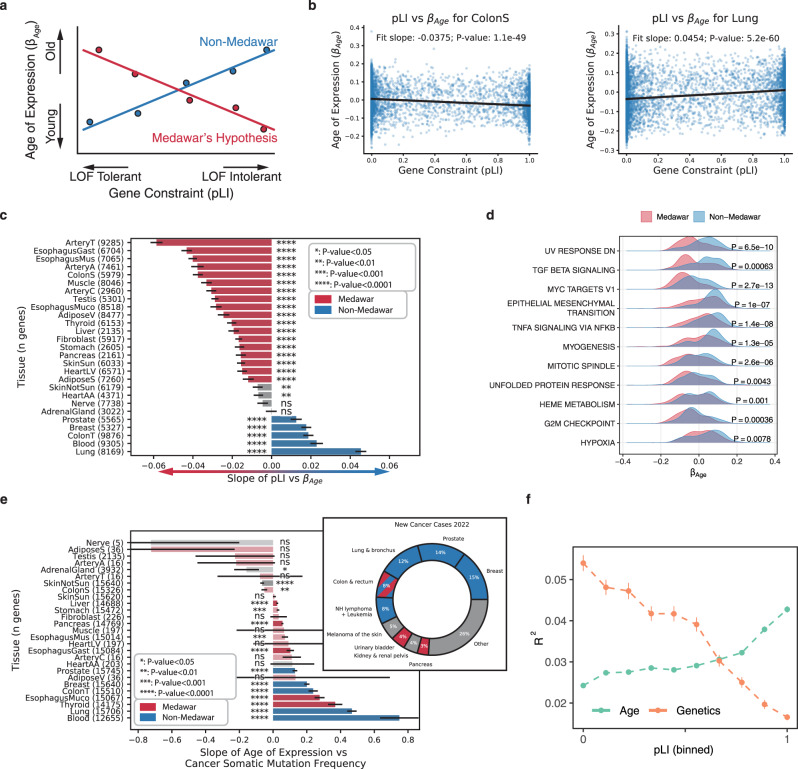
Tissue-specific evolutionary signatures of aging. **a** The expected relationship across genes between the per-gene age-associated slope of gene expression (*β*_age_) and a gene's level of constraint (measured by probability loss of function intolerance, pLI). Medawar's hypothesis predicts a negative relationship (shown in red) between the time of expression and the level of constraint. The opposite trend (non-Medawar) is shown in blue. **b** *β*_age_ across genes plotted as a function of pLI for a tissue exhibiting a *Medawarian* signature, and a *non-Medawarian* signature. **c** The slope of the relationship from **b** between *β*_age_ and constraint across all tissues. Error bars show estimated standard error of regression slope. Unadjusted *p*-values in **b** and **c** are calculated using a linear model two-sided t-test (exact *p*-values in Supplementary Table 2). **d** Hallmark pathways in which the *β*_age_ was significantly different between *Medawarian* and *non-Medawarian* tissues (two-sided *t*-test). **e** Bar chart shows per-tissue relationship between *β*_age_ and frequency of somatic mutations in tumor samples for a particular gene and cancer type (left). Colored as in 5C with alpha indicating unadjusted significance using two-sided *t*-test (exact *p*-values in Supplementary Table 3). Y-axis labels show the number of independent genes and error bars indicate the estimated standard error of regression slope. Doughnut chart shows estimated proportion of new cases of cancer in US in 2022 by cancer type from Table 1 of^25^ (right). Crosshatch indicates that while colon transverse was identified as a non-Medawar tissue, colon sigmoid was not. **f** Gene expression variance explained by genetics or age as a function of binned (10 bins) gene constraint averaged across all tissues. Points represent the mean and error bars the standard error of the mean.

Evolutionary theory predicts that due to the increased impact of selection in younger individuals, genes that increase expression as a function of age (*β*_age_ > 0) should be under reduced selective constraint compared to genes that are highly expressed in young individuals (*β*_age_ < 0), a theory of aging known as *Medawar’s hypothesis*^1^ (Fig. 5a). Several recent studies have demonstrated the generality of this trend across species^8,9,21^, however the tissue-specificity of this theoretical prediction has not been explored. We sought to test the generality of this trend across different tissues by comparing *β*_age_ with the level of constraint on genes, quantified as the probability loss of function intolerance (pLI) score from gnomAD^22^. As expected, across the vast majority of tissues *β*_age_ was significantly negatively correlated with pLI (Fig. 5b, c, Supplementary Fig. 30), in line with Medawar’s hypothesis. However, five tissues exhibited significant signatures in the opposite direction including prostate, transverse colon, breast, whole blood, and lung tissue (*P* < 10^−3^ linear model two-sided *t*-test). These five tissues still maintained a significant negative relationship after subsetting to genes that are highly dependent on age (${R}_{Age}^{2} \; > \;0.1$, Supplementary Fig. 31). These tissues with *non-Medawarian* trends are driven by highly constrained, functionally important genes being expressed at a higher rate in older individuals (Supplementary Fig. 32). Using *dN*/*dS*^23^ as an alternative metric of gene constraint yielded highly correlated results (*R* = −0.72, *P* = 2.5e-5 two-sided Pearson correlation test, Supplementary Figs. 33, 34).

To explore why these five tissues might exhibit distinctive evolutionary signatures of aging we compared the distribution of significant *β*_age_ parameters between *Medawarian* and *non-Medawarian* tissues among different *hallmark pathways*^24^. We found 11 signatures exhibiting significantly increased *β*_age_ (FDR < 0.01 two-sided t-test) compared to *non-Medawarian* tissues (Fig. 5d, Supplementary Fig. 35) including DNA-damage, TGF-*β* signalling, MYC targets, and epithelial-to-mesenchymal transition pathways most prominently. All of these signatures are broadly correlated with cellular proliferation, differentiation, and cancer. Indeed, these five *non-Medawarian* tissues are also the top five most commonly diagnosed sites for cancer in 2022^25^ (Fig. 5e). To directly investigate cancer signatures in these tissues we quantified the per-gene likelihood of having somatic mutations in tumors using the COSMIC cancer browser^26^. GTEx tissues were matched to most representative cancer types for comparisons (e.g. Breast Cancer → Breast Mammary Tissue, Supplementary Data 6). We found that the per-gene age of expression (*β*_*age*_) was significantly correlated with mutation frequency (i.e. mutational burden) across several tissues (Fig. 5e, Supplementary Fig. 36) with the 5 *non-Medawarian* tissues exhibiting some of the strongest signatures (*P* < 10^−4^ linear model two-sided *t*-test). These results highlight that gene expression patterns in tissues and cell-types that proliferate throughout the course of an individuals life may be subjected to distinct evolutionary pressures with important implications for the cancer susceptibility of these tissues.

We also explored the relationship between gene expression heritability and constraint. Across all tissues *h*^2^ was significantly negatively correlated with pLI (*P*-value < 10^−3^ linear model two-sided t-test, Supplementary Figs. 37, 38, 39). Thus, on average genes in which the variation in expression levels is heritable tend to be under significantly less functional constraint (Fig. 5f). In contrast however, on average ${R}_{age}^{2}$ increases as function of pLI, highlighting the increased constraint of many of the genes that exhibit age-associated changes in gene expression. These highly conserved genes (e.g. the aforementioned mitochondrial and translation factors) are thus potentially of critical importance to disease. Together, these results highlight the stark contrast in the types of genes with heritable expression patterns (reduced constraint) compared to those with age-associated gene expression patterns (increased constraint).

## Discussion

Studying age-associated changes in gene expression provides critical insights into the underlying biological processes of aging. Here, we set out to quantify the relative contributions of aging and genetics to gene expression phenotypes across different human tissues. Our study finds that the predictive power of eQTLs is significantly impacted by age across several different tissues and that this effect is more pronounced in older individuals. These results extend upon previous work examining blood tissue^4^ and highlight the varied impact of aging on eQTLs among different tissues. We show that this result is likely to be in part due to an increase in the inter-individual heterogeneity of gene expression patterns among individuals in some contexts, potentially as a result of the increased impact of the environment. Notably, increased inter-individual heterogeneity in both younger and older individuals was associated with reduced predictive power of eQTLs as well as expression heritability. This relationship is expected as an increase in gene expression heterogeneity would reduce the proportion of heritable gene expression. Our study was not able to determine why the inter-individual heterogeneity in gene expression might differ between old and young individuals, regardless of the direction of the effect. Potentially different tissues are subjected to varying contributions of the environment at different ages, however, testing such a hypothesis in humans is challenging.

When testing for individual genes with age-associated heteroskedasticity we found few significant genes. Furthermore, most of the heteroskedasticity identified was negative, i.e. exhibiting decreases in variance associated with age. In contrast however, our analyses considering the transcriptome of each individual as a whole identified several instances of increased inter-individual variation (Fig. 2). This could potentially result from the combined effects of many small changes in expression variance across genes, not individually detectable given the current sample sizes. Alternatively, unique, individual-specific changes in gene expression, potentially due to unique environments could increase the variance of the transcriptome as a whole between individuals. Future work may help distinguish between these hypotheses.

Our study is however limited in its primary focus on bulk-tissue transcriptomic data. Early evidence from single cell studies already suggests that differences in gene expression heterogeneity vary among cell types of tissues as a function of age^6,7,27,28^. While these studies lack sufficient individual sample sizes and genetic diversity for the statistical approaches used herein, it is possible that in the future the availability of larger datasets will facilitate studying these phenomena at the single-cell level. The extensive tissue heterogeneity we observe suggests that patterns of aging will exhibit substantial cell-type specificity.

We also present an approach to jointly model the impact of genetics and aging on gene expression variance to parse out the individual contributions of each of these factors. The increased complexity of our model has little impact on its accuracy with our expression heritability estimates strongly correlated with previous heritability measures across all tissues (mean Pearson’s *r* = 0.89, Supplementary Fig. 20). Using this model we show that age exhibits exceptionally varied affects on different tissues, and indeed, in several tissues age contributes more to gene expression variance on average than genetics. These results also highlight a widespread coordinated signature of age-associated decline in mitochondrial and translation factors. Dysregulation in mitochondrial function and ribosome biogenesis have been documented as key players in aging^29,30^, however our results highlight the tissue-specificity of these trends. Our model also allows us to quantify the tissue-specific evolutionary context of age-associated gene expression changes. We corroborate the inverse relationship between age-at-expression and constraint, as predicted by Medawar’s hypothesis and recently documented by others^8,9,21^ across the vast majority of tissues. However, we also surprisingly identify five tissues which exhibit the opposite pattern and show that age-associated signatures of increased proliferation and cancer are enriched in these tissues. These results highlight the distinct evolutionary forces that act on late-acting genes expressed in highly proliferative cell-types. Future work extending these analyses to the single-cell level will provide further insights into the cell-type-specific age-associated patterns of constraint, and its relevance to cancer.

Overall this work has several important implications. Our results shed light on recent work on the prediction accuracy of polygenic risk scores (PRS)^31^ which found that numerous factors, including age, sex, and socioeconomic status can profoundly impact the prediction accuracy of such scores even in individuals with the same genetic ancestry. Our results highlight that genetics exhibit varied predictive power in several different tissues as a function of age, potentially playing a role in differential PRS accuracy between young and old individuals. This also has important implications for disease association and prediction approaches that leverage expression quantitative trait loci to prioritize variants, including colocalization methods^32^, transcriptome-wide association studies^14,33^, and Mendelian randomization^34,35^. If a significant proportion of eQTLs exhibit age-associated biases in their effect size in a tissue of interest, then these approaches may be less powerful when applied to diseases for which age is a primary risk factor such as heart disease, Alzheimer’s dementias, cancers, and diabetes. Furthermore our results highlight that genes with eQTLs tend to be subject to less evolutionary constraint, and thus potentially less biologically important, in contrast to genes with age-associated gene expression patterns which exhibit increased constraint.

The critical role of aging as a risk factor for many common human diseases underscores the importance of understanding its impact on cellular systems at the molecular level. Together our analyses provide insights into tissue-specific patterns of aging and the relative impact of genetics and aging on gene expression. We anticipate that future studies across tissues and cells of gene expression, chromatin structure, and epigenetics will further elucidate how both programmed and stochastic processes of aging drive human disease.

## Methods

### Data collection age groupings

We downloaded gene expression data for multiple individuals and tissues from GTEx V8^10^, which were previously aligned and processed against the hg19 human genome. Tissues were included in the analysis if they had >100 individuals in both the age ≥55 and <55 cohorts (Supplementary Fig. 2). For a given tissue, genes were included if they had >0.1 TPM in ≥20% of samples and ≥6 reads in ≥20% of samples, following GTEx’s eQTL analysis pipeline. To compare gene expression heritability across individuals of different ages, for some analyses we split the GTEx data for each tissue into two age groups, "young" and "old," based on the median age of individuals in the full dataset, which was 55 (Supplementary Fig. 1). Within each tissue dataset, we then equalized the number of individuals in the young and old groups by randomly downsampling the larger group, to ensure that our models were equally powered for the two age groups.

### PEER factor analysis

We analyzed existing precomputed PEER factors available from GTEx to check for correlations between these hidden covariates and age. In particular, we fit a linear regression between age and each hidden covariate and identified significant age correlations using an F-statistic (Supplementary Fig. 3). Because some of the covariates were correlated with age, we generated age-independent hidden covariates of gene expression to remove batch and other confounding effects on gene expression while retaining age related variation. In particular, we first removed age contributions to gene expression by regressing gene expression on age and then ran PEER on the age-independent residual gene expression to generate 15 age-independent hidden PEER factors.

### Quantifying the effect of eQTLs on gene expression in different age groups

Using the binary age groups defined above, we assessed the relative significance of eQTLs in old and young individuals by carrying out separate assessment of eQTLs identified by GTEx. We report the number of genes included in analysis for each tissue (Supplementary Table 1). For each gene in each tissue and each age group, we regressed the GTEx pre-normalized expression levels on the genotype of the lead SNP (identified by GTEx, MAF > 0.01) using 5 PCs, 15 PEER factors, sex, PCR protocol and sequencing platform as covariates, following the GTEx best practices. We confirmed our results using both our recomputed PEER factors as well as the PEER factors provided by GTEx (Supplementary Fig. 5). To test for significant differences in genetic associations with gene expression between the old and young age groups, we compared the p-value distributions between these groups for all genes and all SNPs in a given tissue using Welch’s t-test. To investigate the validity of the age cutoff used for these binary age groups, we replicated the eQTL analysis using two additional age cutoffs of 45 and 65 years old. We observed the same trends in both cases; however, statistical power decreased due to smaller sample sizes in the resulting age bins, leading to a non-significant result for age cutoff 45 (Supplementary Fig. 40).

### Jensen-Shannon Divergence as a distance metric between transcriptome profiles

To quantify differences in gene expression between individuals, we computed the pairwise distance for all pairs of individuals in an age group using the square root of Jensen-Shannon Divergence (JSD) distance metric, which measures the similarity of two probability distributions. Here we applied JSD between pairs of individuals’ transcriptome vectors containing the gene expression values for each gene, which we converted to a distribution by normalizing by the sum of the entries in the vector. For two individuals’ transcriptome distributions, the JSD can be calculated as:

1 $${{{{{{{\rm{JSD}}}}}}}}({P}_{1},\;{P}_{2})=H\left(\frac{1}{2}{P}_{1}+\frac{1}{2}{P}_{2}\right)-\frac{1}{2}(H({P}_{1})+H({P}_{2}))$$

where *P*_*i*_ is the distribution for individual *i* and H is the Shannon entropy function:

2 $$H(X)=-\mathop{\sum }\limits_{i=1}^{n}P({x}_{i}){\log }_{2}(P({x}_{i}))$$

JSD is known to be a robust metric that is less sensitive to noise when calculating distance compared to traditional metrics such as Euclidean distance and correlation. It has been shown that JSD metrics and other approaches yield similar results but that JSD is more robust to outliers^12^. The square root of the raw JSD value follows the triangle inequality, enabling us to treat it as a distance metric.

### Slope of JSD distance versus age

In addition to comparing JSD between the two age groups defined above, "young" and "old", we also binned all GTEx individuals into 6 age groups, from 20 to 80 years old with an increment of 10 years. We then computed pairwise distance and average age for each pair of individuals within each bin using the square root of JSD as the distance metric. We applied a linear regression model of JSD versus age to obtain slopes, confidence intervals, and p-values.

### Cell-type specific analysis

To analyze whether cell type composition affects age-associated expression changes, we utilized the tool CIBERSORTx^16^ to estimate cell type composition and individual cell type expression levels in GTEx whole blood. Cell type composition estimates were computed using CIBERSORTx regular mode. Individual cell type expression level estimates were computed using CIBERSORTx high resolution mode. We then repeated our JSD and eQTL analyses on each cell type independently (see JSD and eQTL sections for details). In addition, to analyze tissue-specific differences in cell type composition, we referred to a previous study^36^ that computed cell type composition for different GTEx tissues using CIBERSORTx. We applied the JSD metric to each tissue, using the cell type composition vector as the distribution. Additionally, we applied the Breusch-Pagan test to compute heteroskedasicity coefficients and p-values with respect to age, after inverse logit transformation to give an approximately Gaussian distribution (Supplementary Fig. 44) (see section on heteroskedastic gene expression).

### Heteroskedastic gene expression

We used the Breusch-Pagan test to call heteroskedastic gene expression with age. For each gene and tissue, we computed gene expression residuals by regressing out age-correlated PEER factors, other GTEx covariates, and age. To test for age-related heteroskedasticity, we squared these residuals and divided by the mean, regressed them against age, and looked at the age effect size (*β*_*het*_). We called significantly heteroskedastic genes using a two-sided t-test with the null hypothesis that the *β*_*het*_ is zero. The Benjamini-Hochberg procedure was used to control for false positives. To determine which tissues have more genes with increasing gene expression heterogeneity with age, we compare the number of genes with positive heteroskedasticity (*β*_*het*_ > 0 and *FDR* < 0.2) to the total of all heteroskedastic genes (*FDR* < 0.2). We compare this metric to the per-tissue 2-bin JSD (Supplementary Fig. 41) and 6-bin JSD slope (Supplementary Fig. 15).

### Multi-SNP gene expression prediction

We used a multi-SNP gene expression prediction model based on PrediXcan^14^ to corroborate our findings from the eQTL and JSD analyses on the two age groups, "young" and "old". For each gene in each tissue, we trained a multi-SNP model separately within each age group to predict individual-level gene expression.

3 $${Y}_{g,t}=\mathop{\sum}\limits_{i}{\beta }_{i,g,t}{X}_{i}+\epsilon$$

Where *β*_*i*,*g*,*t*_ is the coefficient or effect size for SNP *X*_*i*_ in gene *g* and tissue *t* and *ϵ* includes all other noise and environmental effects. The regularized linear model for each gene considers dosages of all common SNPs within 1 megabase of the gene’s TSS as input, where common SNPs are defined as MAF > 0.05 and Hardy-Weinberg equilibrium *P* > 0.05. We removed covariate effects on gene expression prior to model training by regressing out both GTEx covariates and age-independent PEER factors (described above). Coefficients were fit using an elastic net model which solves the problem^37^:

4 $${\min }_{\beta_{0},\;\beta }\frac{1}{2N}\mathop{\sum }\limits_{j=1}^{N}{\left({Y}_{j}-{\beta }_{0}-{X}_{j}^{T}\beta \right)}^{2}+\lambda \left(\frac{1-\alpha }{2}||\beta|{|}_{2}^{2}+\alpha||\beta|{|}_{1}\right)$$

The minimization problem contains both the error of our model predictions ${({Y}_{j}-{\beta }_{0}-{X}_{j}^{T}\beta )}^{2}$ and a regularization term $\lambda (\frac{1-\alpha }{2}||\beta|{|}_{2}^{2}+\alpha||\beta|{|}_{1})$ to prevent model overfitting. The elastic net regularization term incorporates both L1 (∣∣*β*∣∣_1_)) and L2 ($||\beta|{|}_{2}^{2}$) penalties. Following PrediXcan, we weighted the L1 and L2 penalties equally using *α* = 0.5^14^. For each model, the regularization parameter *λ* was chosen via 10-fold cross validation. The elastic net models were fit using Python’s glmnet package and *R*^2^ was evaluated using scikit-learn. From the trained models for each gene, we evaluated training set genetic *R*^2^ (or *h*^2^) for the two age groups and subtracted ${h}_{{{{{young}}}}}^{2}-{h}_{{{{{old}}}}}^{2}$ to get the difference in gene expression heritability between the groups. We compared this average difference in heritability to the mean JSD_*old*_ − JSD_*young*_ and $\log ({P}_{old})-\log ({P}_{young})$ using *P*-values from the eQTL analyses across genes.

### Joint model for expression prediction using SNPs and age

To uncover linear relationships between gene expression and both age and genetics, we built a set of gene expression prediction models using both common SNPs and standardized age as input. An individual’s gene expression level *Y* for a gene *g* and tissue *t* is modeled as:

5 $${Y}_{g,t}=\mathop{\sum}\limits_{i}{\beta }_{i,g,t}{X}_{i}+{\beta }_{{{{{{{{\rm{age}}}}}}}},g,t}A+\epsilon$$

Where *A* is the normalized age of an individual. Coefficients were fit using elastic net regularization, as above, which sets coefficients for non-informative predictors to zero. The sign of the fitted age coefficient (*β*_*age*,*g*,*t*_), when nonzero, reflects whether the gene in that tissue is expressed more in young (negative coefficient) or old (positive coefficient) individuals. We also evaluated the training set *R*^2^ using the fit model coefficients separately for genetics (across all SNPs in the model) and age:

6 $${R}_{genetics}^{2}={h}^{2}={R}^{2}({Y}_{g,t},\mathop{\sum}\limits_{i}{\beta }_{i,g,t}{X}_{i})$$

7 $${R}_{age}^{2}={R}^{2}({Y}_{g,t},\;{\beta }_{{{{{{{{\rm{age}}}}}}}},g,t}A)$$

We also tested whether the age-related gene expression relationship was sex-specific by rerunning the joint model with an additional age-sex interaction term as follows:

8 $${Y}_{g,t}=\mathop{\sum}\limits_{i}{\beta }_{i,g,t}{X}_{i}+{\beta }_{{{{{{{{\rm{age}}}}}}}},g,t}A+{\beta }_{{{{{{{{\rm{age}}}}}}}} * {{{{{{{\rm{sex}}}}}}}},g,t}A * S+\epsilon$$

Where *β*_age∗sex,*g*,*t*_ is the additional model weight for the age-sex interaction term and *S* is the binary sex of the GTEx individual. The *R*^2^ of age, genetics, and the age-sex interaction term are evaluated as before by determining the variance explained by each term in the model. We compared the ${R}_{age}^{2}$ between the models including or excluding the age-sex interaction term (Supplementary Fig. 26). We also compared the tissue-averaged variance explained by age and the age-sex interaction term. Finally, to check the consistency of tissue-specific gene expression heritability estimates from our model and the original PrediXcan model trained on GTEx data, we evaluate Pearson’s r between our heritability estimates and those of PrediXcan (Supplementary Fig. 20), using heritability estimates from the original PrediXcan model available in PredictDB.

### Tissue specificity of age and genetic associations

We evaluated the variability of age and genetic associations across tissues using a measure of tissue specificity for age and genetic *R*^2^^38^. We measured the tissue-specificity of a gene *g*’s variance explained ${R}_{g}^{2}$ using the following metric:

9 $${S}_{g}=\frac{\mathop{\sum }\nolimits_{t=1}^{n}\left(1-\frac{{R}_{g,t}^{2}}{{R}_{g,\max }^{2}}\right)}{n-1}$$

Where *n* is the total number of tissues, ${R}_{g,t}^{2}$ is the variance explained by either age or genetics for the gene *g* in tissue *t* and ${R}_{g,\max }^{2}$ is the maximum variance explained for *g* over all tissues. This metric can be thought of as the average reduction in variance explained relative to the maximum variance explained across tissues for a given gene. The metric ranges from 0 to 1, with 0 representing ubiquitously high genetic or age *R*^2^ and 1 representing only one tissue with nonzero genetic or age *R*^2^ for a given gene. We calculate *S*_*g*_ separately for ${R}_{{{{{{{{\rm{age}}}}}}}}}^{2}$ and ${R}_{{{{{{{{\rm{genetics}}}}}}}}}^{2}$ across all genes.

### Functional constraint analysis

We quantified gene constraint using the probability of loss of function intolerance (pLI) from gnomAD 2.1.1^22^. We analyzed the relationships between pLI vs *β*_age_ and pLI vs heritability across genes. For these analyses, genes were only included if age or genetics were predictive of gene expression (*R*^2^ > 0) for that gene. For genes with *R*^2^ > 0, we used linear regression to determine the direction of the relationship between pLI and *β*_age_ or heritability for each tissue. The F-statistic was used to determine whether pLI was significantly related to these two model outputs. For pLI vs *β*_age_, a significant negative slope was considered a *Medawarian* trend (consistent with Medawar’s hypothesis) and a significant positive slope a *non-Medawarian* trend. To test whether the non-Medawarian trends were driven by genes with higher expression, we excluded genes in the top quartile of median gene expression and repeated the analysis between pLI and *β*_age_ (Supplementary Fig. 42). We also analyzed the evolutionary constraint metric dN/dS^23^ and its tissue-specific relationship with *β*_age_ by determining the slope and significance of the linear regression, as above.

### Cancer somatic mutation frequency

We quantified the per-gene and per-tissue cancer somatic mutation frequency using data from the COSMIC cancer browser^26^. For each tissue, we selected the closest cancer type as noted in Supplementary Data 5 and downloaded the number of mutated samples (tumor samples with at least one somatic mutation within the gene) and the total number of samples for all genes. We computed the cancer somatic mutation frequency by dividing the number of mutated samples by the total number of samples. For each tissue, we plotted the gene’s *β*_age_ vs its cancer somatic mutation frequency for all genes with >200 tumor samples. We report the slope and significance of the relationship between *β*_age_ and cancer somatic mutation frequency for each tissue. To determine whether age-dependent gene expression heteroskedasticity is related to a gene’s involvement in cancer (Supplementary Fig. 43), we also plotted each gene’s heteroskedasticity effect size vs the cancer somatic mutation frequency for all genes with >200 tumor samples and moderately significant heteroskedasticity (FDR < 0.2). Tissues with ≤5 genes meeting these criteria are not plotted.

### *Non-Medawarian* tissue analysis

To explore the *non-Medawarian* trend in some tissues, we assessed the distribution of *β*_age_ across *Medawarian* and *non-Medawarian* tissues for genes within each of the 50 MSigDB hallmark pathways^24^. Significant differences between the distributions were called using a t-test, and p-values were adjusted for multiple hypothesis testing using a Benjamini-Hochberg correction.

### Reporting summary

Further information on research design is available in the Nature Research Reporting Summary linked to this article.

## Supplementary information

Supplementary Information

Description of Additional Supplementary Files

Supplementary Data 1-6

Reporting Summary

## Data Availability

Processed PEER factors are available on GitHub https://github.com/sudmantlab/gene_expression_aging^39^ and full results for joint age and genetic model can be found on Zenodo 10.5281/zenodo.6555453^40^. The raw GTEx V8 expression data can be obtained at https://gtexportal.org/home/datasets. GTEx V8 genetic data are available under restricted access at https://gtexportal.org/home/protectedDataAccess. The gene expression measurements and genotype for PIVUS cohort is available under European Genome Archive at EGAD00001004965.

## Code availability

All analyses were performed in R version 4.0.2, Python 3.6.13, Numpy 1.19.5, Scikitlearn 0.24.2, Pandas 1.1.5, glmnet 2.2.1, PEER 1.3 and GCTA 1.94.1 CIBERSORTx (https://cibersortx.stanford.edu/). All code is available online at https://github.com/sudmantlab/gene_expression_aging^39^ and archived at 10.5281/zenodo.6555500.
