# Supplementary material for: Tissue-specific impacts of aging and genetics on gene expression patterns in humans
Source: Nat Commun. 2022 Oct 3;13:5803. doi: 10.1038/s41467-022-33509-0 (PMC9530233; doi:10.1038/s41467-022-33509-0)
Supplement: Supplementary file 1 — Supplementary Information [file 41467_2022_33509_MOESM1_ESM.pdf]

Supplementary

Tissue	Number of eGenes
Whole_Blood	20315
Stomach	24290
Colon_Sigmoid	24483
Esophagus_Gastroesophageal_Junction	24168
Colon_Transverse	25379
Artery_Aorta	23959
Heart_Atrial_Appendage	23194
Breast_Mammary_Tissue	25849
Prostate	26529
Heart_Left_Ventricle	21353
Esophagus_Muscularis	23871
Adipose_Visceral_Omentum	24724
Esophagus_Mucosa	23949
Lung	26095
Skin_Not_Sun_Exposed_Suprapubic	25279
Nerve_Tibial	25873
Thyroid	26054
Adipose_Subcutaneous	24665
Artery_Tibial	23304
Testis	35007
Skin_Sun_Exposed_Lower_leg	25196
Muscle_Skeletal	21031
Pancreas	22615
Liver	22262
Artery_Coronary	24529
Adrenal_Gland	23820
Cells_Cultured_fibroblasts	22050

Supplementary Table 1. Number of GTEx eGenes analyzed per tissue

Tissue	Slope of β_{Age} vs pLI	P-value
ArteryT	-0.0585	5.7e-80
EsophagusGast	-0.0432	3.0e-58
EsophagusMus	-0.0401	1.1e-75
ArteryA	-0.0377	7.8e-33
ColonS	-0.0375	1.1e-49
Muscle	-0.0331	1.8e-34
ArteryC	-0.0306	1.0e-33
Testis	-0.0288	2.6e-55
EsophagusMuco	-0.0283	2.2e-23
AdiposeV	-0.0244	9.5e-17
Thyroid	-0.0203	3.1e-18
Liver	-0.0193	6.8e-13
Fibroblast	-0.0167	4.7e-22
Stomach	-0.0163	1.2e-11
Pancreas	-0.0157	9.5e-10
SkinSun	-0.0156	3.3e-14
HeartLV	-0.0149	1.1e-09
AdiposeS	-0.0119	1.8e-05
SkinNotSun	-0.0070	4.9e-03
HeartAA	-0.0067	4.9e-03
Nerve	-0.0045	9.5e-02
AdrenalGland	-0.0001	9.6e-01
Prostate	0.0125	9.2e-06
Breast	0.0177	1.9e-12
ColonT	0.0187	4.1e-13
Blood	0.0229	4.2e-13
Lung	0.0454	5.2e-60

Supplementary Table 2. Slope and p-values of relationship between pLI and β_{Age} from Fig 5c for all tissues.

Tissue	Slope of β_{Age} vs Cancer Mutation Frequency	P-value
Nerve	-0.7244	2.6e-01
AdiposeS	-0.7224	1.5e-01
Testis	-0.2255	3.4e-01
ArteryA	-0.2185	3.6e-01
AdrenalGland	-0.1574	3.2e-02
ArteryT	-0.0777	7.6e-01
SkinNotSun	-0.0645	9.1e-09
ColonS	-0.0517	2.3e-03
SkinSun	0.0079	3.7e-01
Liver	0.0245	1.8e-05
Stomach	0.0291	1.5e-04
Fibroblast	0.0329	5.0e-01
Pancreas	0.0555	6.6e-22
Muscle	0.0650	8.2e-01
EsophagusMus	0.0705	1.4e-04
HeartLV	0.0910	6.6e-01
EsophagusGast	0.1026	1.5e-06
ArteryC	0.1110	5.9e-02
HeartAA	0.1143	3.8e-01
Prostate	0.1297	1.7e-21
AdiposeV	0.1325	8.1e-01
Breast	0.2004	3.6e-40
ColonT	0.2375	5.9e-18
EsophagusMuco	0.2757	6.8e-21
Thyroid	0.3683	2.6e-18
Lung	0.4674	2.9e-63
Blood	0.7484	4.9e-11

Supplementary Table 3. Slope and p-values of relationship between β_{Age} vs Cancer Somatic Mutation Frequency from Fig 5e for all tissues.

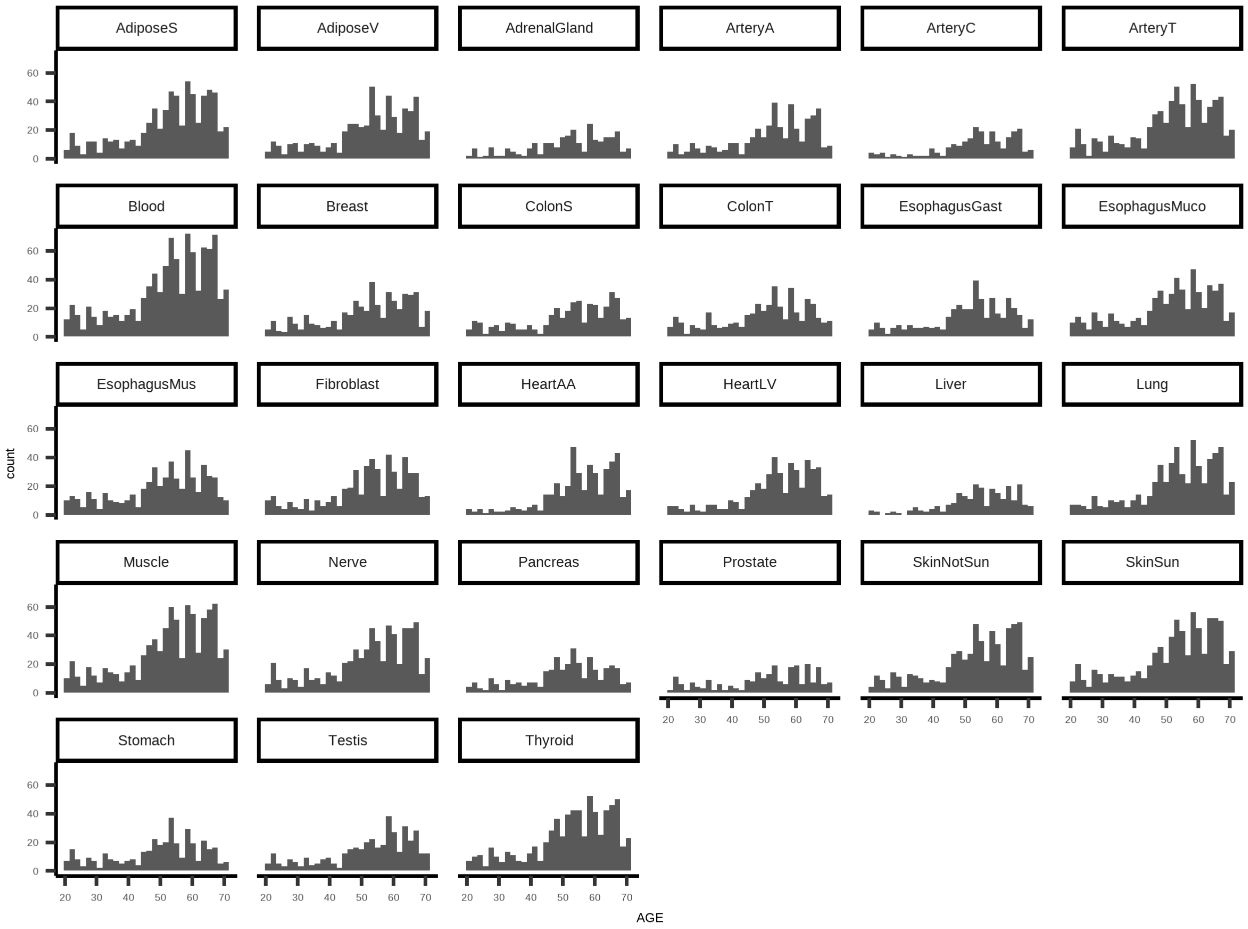

Supplementary Figure 1. Histogram representing age distribution of each GTEx tissue, x-axis representing age of each sample and y-axis representing counts in bin

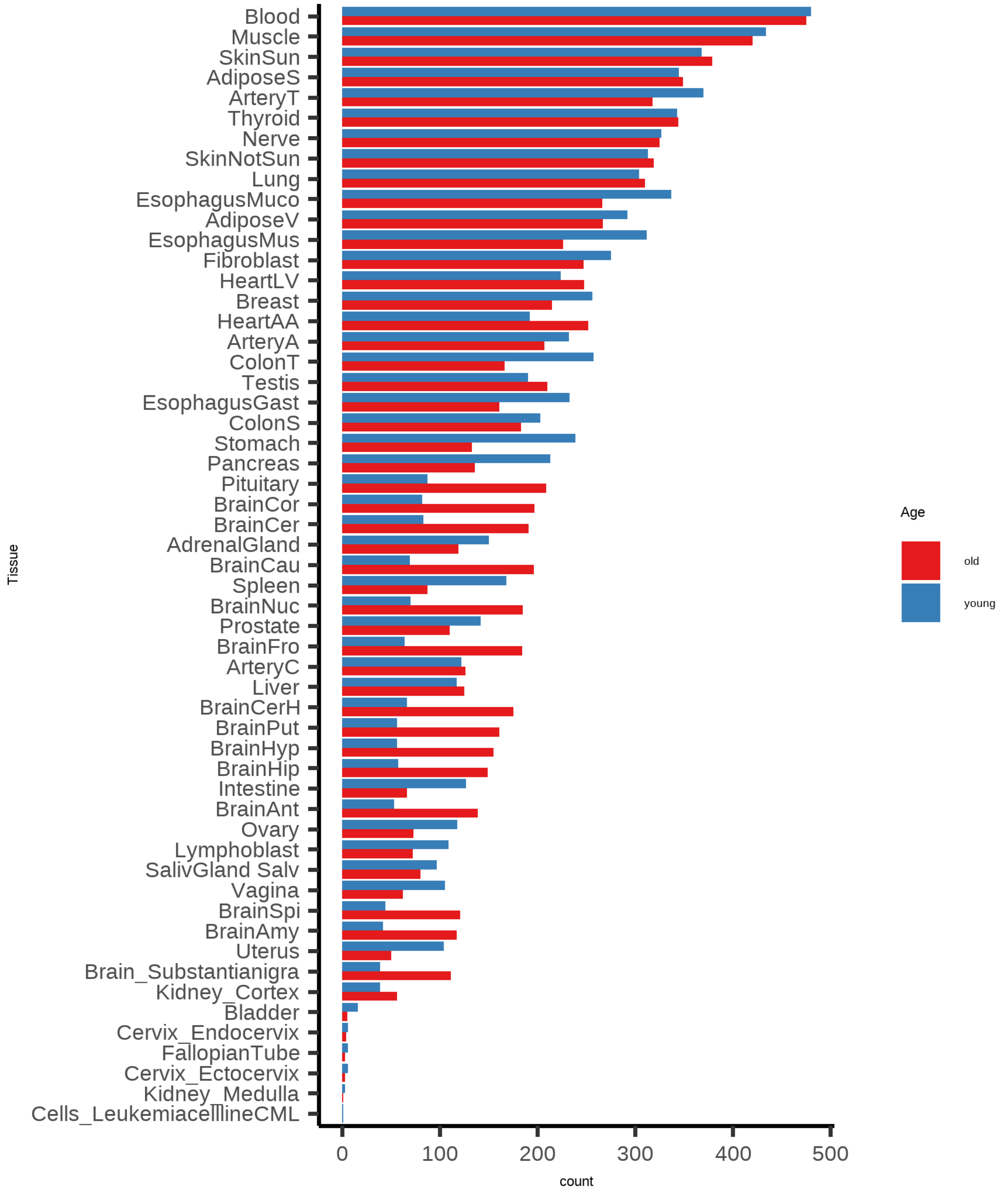

Supplementary Figure 2. Number for individuals above and below the median age of 55 by tissue for all 47 GTEx tissues

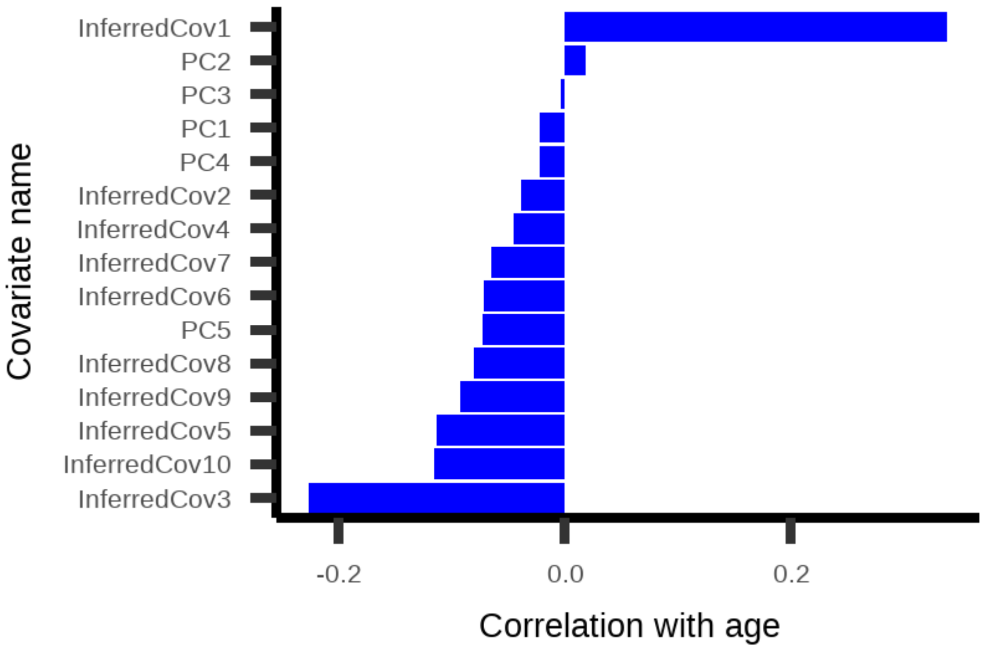

Supplementary Figure 3. Pearson's r between GTEx covariates with age. PEER factor covariates labeled as "InferredCov" and genetic principle components labeled as "PC"

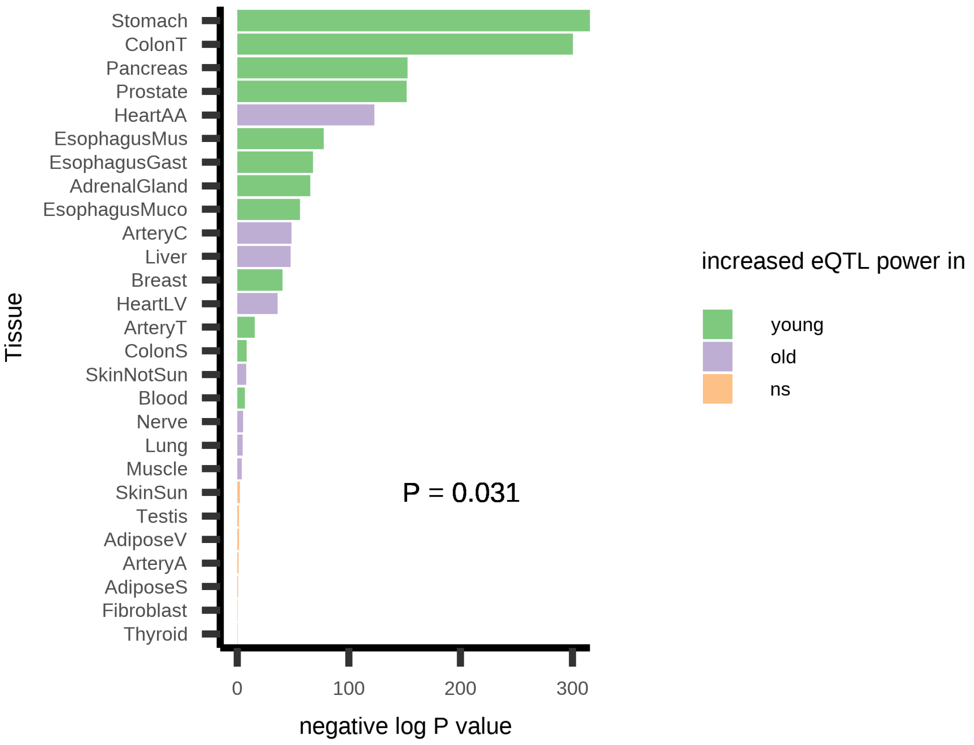

Supplementary Figure 4. Barplot representing the negative log p-value of the difference in eQTL p-value distribution between old and young individuals for each tissue (Fig. 1C). Color code represents whether eQTL power was increased in young, old, or neither (no significant difference). P-value of difference in eQTL power is obtained from two-sided Wilcoxon rank sum test. Note that negative log p-value for Stomach is infinity, however the bar plot has been trimmed for visualization purposes.

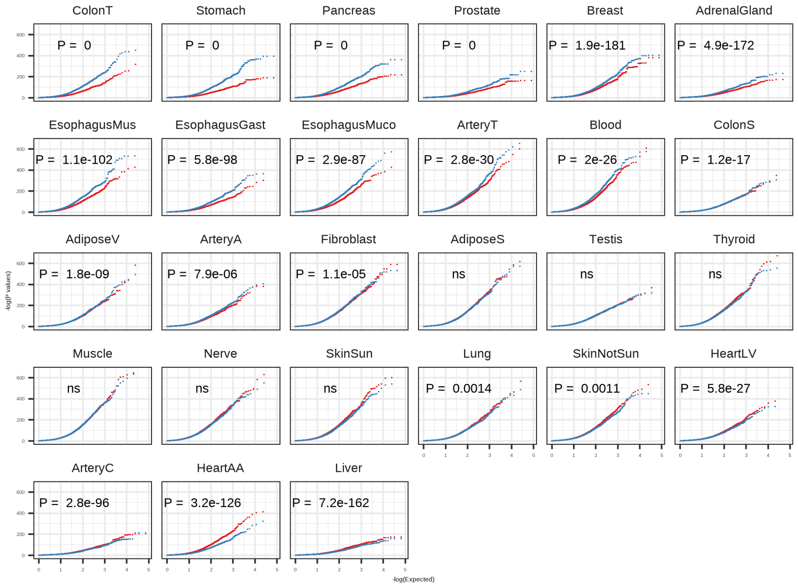

Supplementary Figure 5. QQ plot of eQTL p-values for old (red) and young (blue) cohorts across 27 tissues using GTEx PEER factors. Significant differences between p-value distributions annotated for each tissue. P-values are obtained from two-sided Welch's t test.

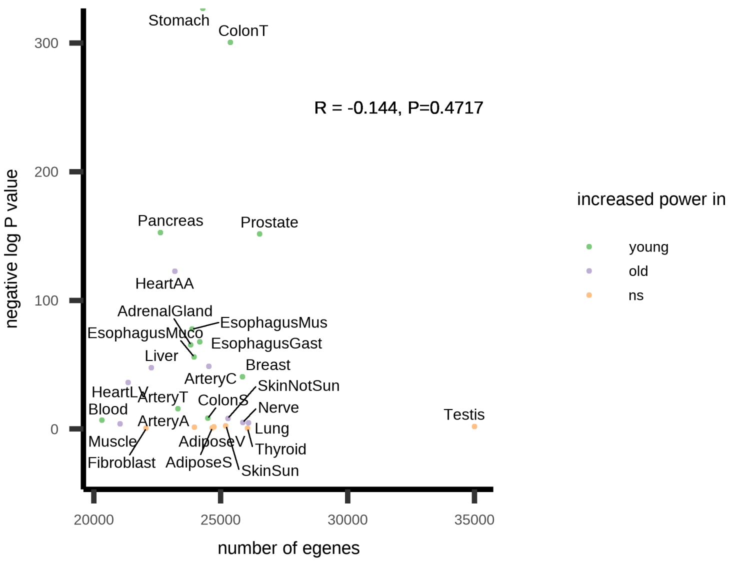

Supplementary Figure 6. Scatter plot of per-tissue number of GTEx eGenes and the difference in eQTL power between old and young individuals. P-values of relationship determined using two-sided spearman correlation test.

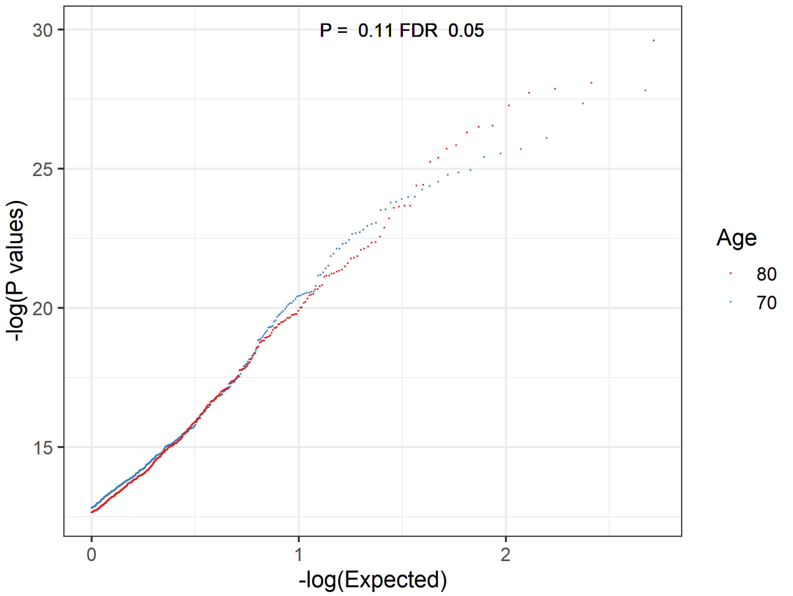

Supplementary Figure 7. QQ plot of eQTL p-values from individuals of age 70 and 80 from PIVUS cohort using genes under FDR cutoff of 0.05. P-values for distribution difference are obtained from two-sided Welch's t test.

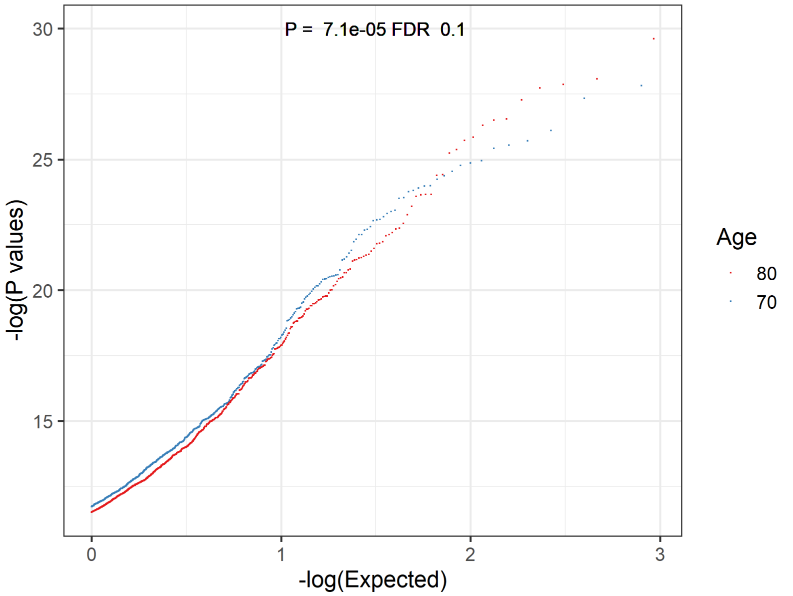

Supplementary Figure 8. QQ plot of eQTL p-values from individuals of age 70 and 80 from PIVUS cohort using genes under FDR cutoff of 0.1. P-values for distribution difference are obtained from two-sided Welch's t test.

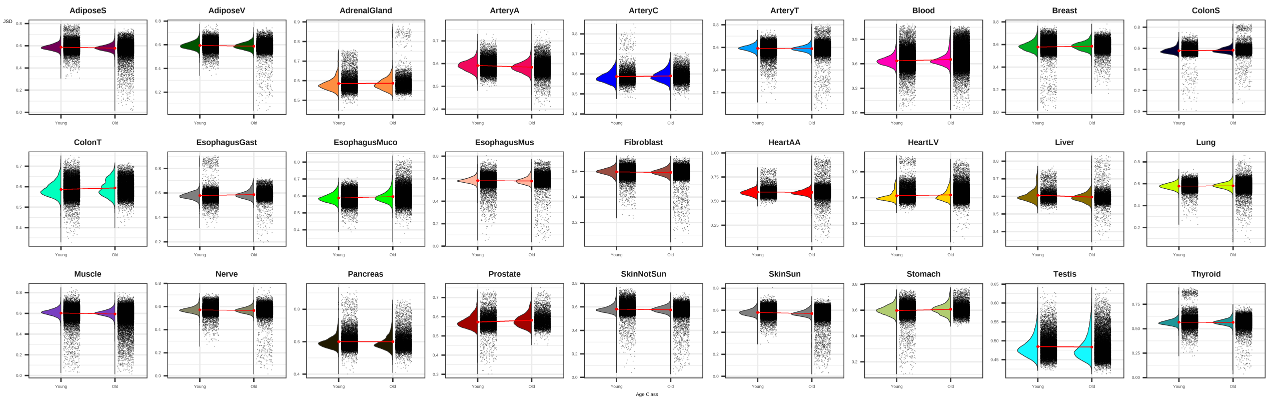

Supplementary Figure 9. The distributions of JSD distances for all tissues in old and young bins.

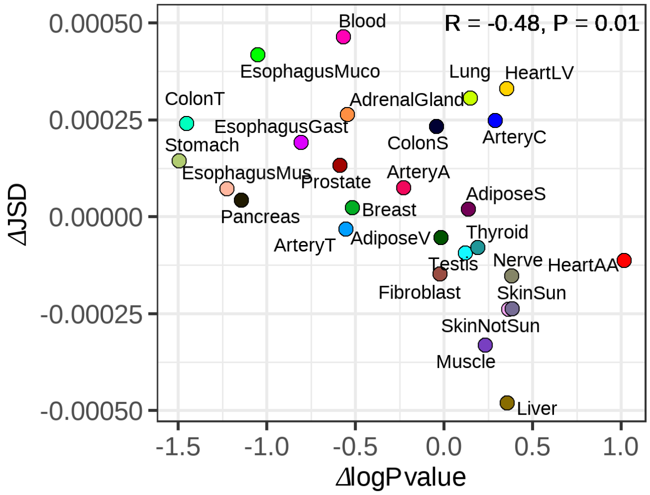

Supplementary Figure 10. Scatter plot showing the correlation between the difference in average JSD between young and old individuals and difference in eQTL log(p-value) between young and old. R and p-value are obtained from two sided Pearson correlation test.

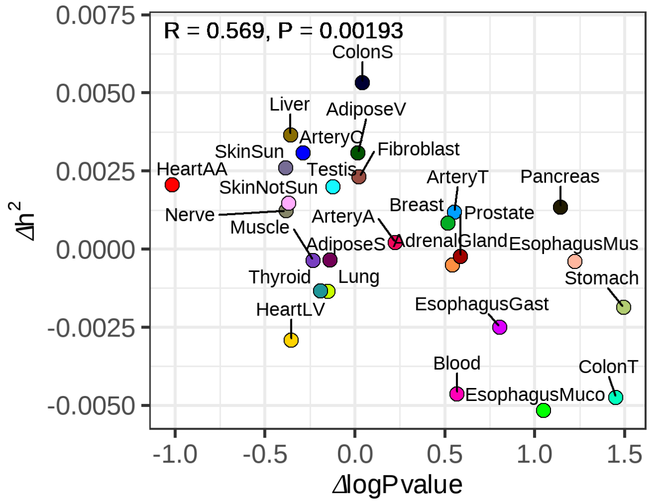

Supplementary Figure 11. High correspondence of age-related changes in gene expression associations for single SNP and multi-SNP models. Scatter plot showing the correlation between the difference in heritability estimated by the multi-SNP linear model (PrediXcan) in young and old individuals and difference in eQTL log(p-value) in young and old individuals.R and p-value are obtained from two-sided Pearson correlation test.

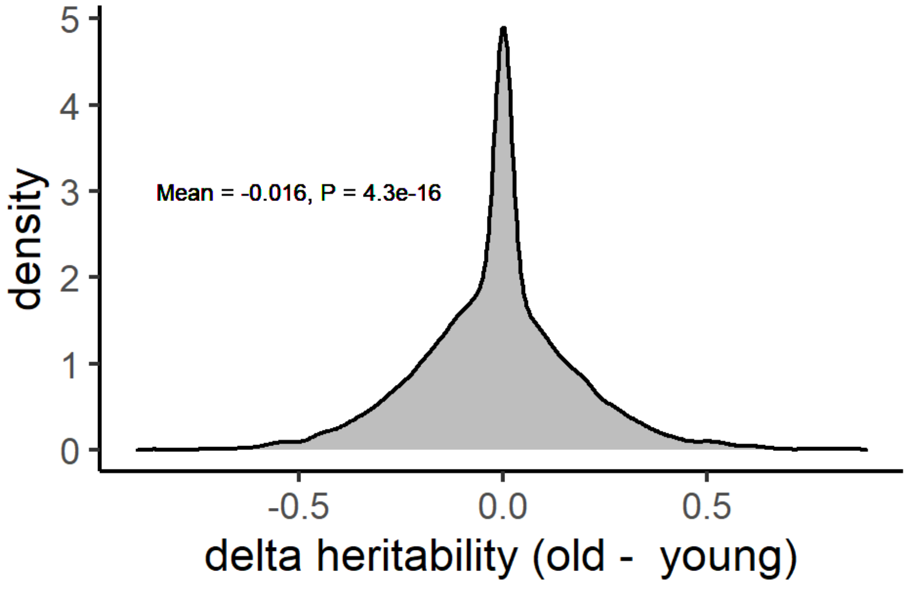

Supplementary Figure 12. Distribution of Δh^2 (80 - 70) in PIVUS cohort. P-value is obtained from two-sided one-sample t-test.

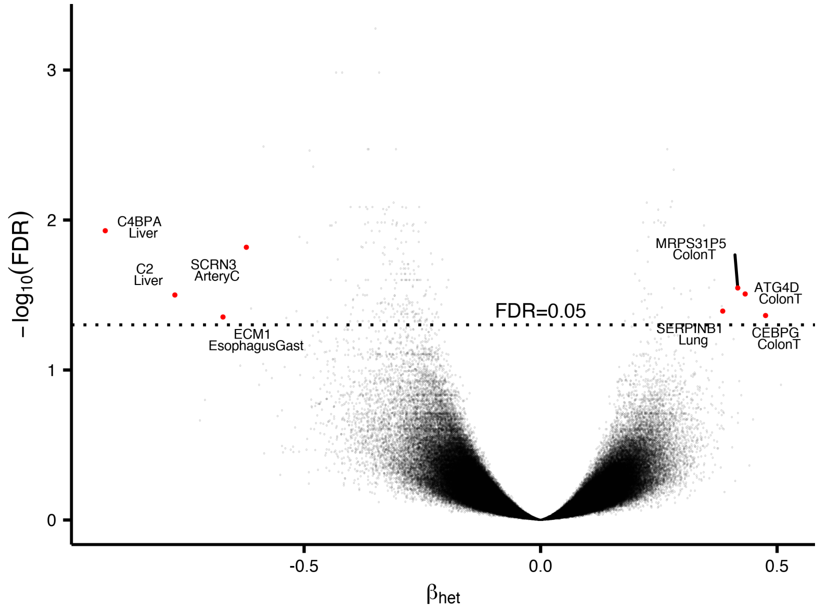

Supplementary Figure 13. Volcano plot of the age-related heteroskedasticity significance vs effect size for each tested gene's expression. FDR cutoffs of 0.05 is indicated. Genes with top 4 largest and smallest effect sizes with significant heteroskedasticity are labeled.

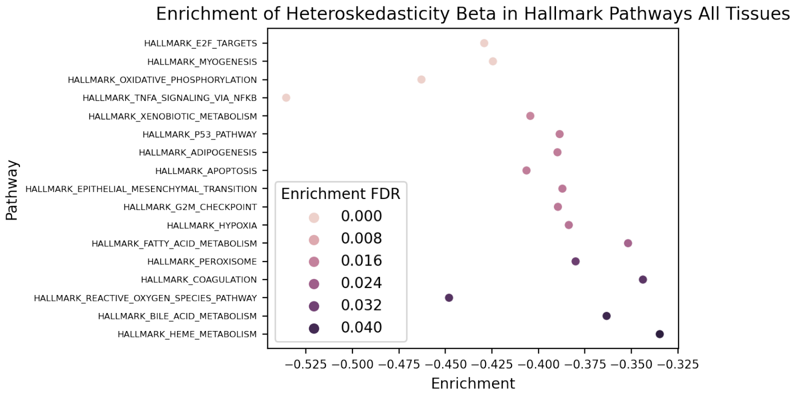

Supplementary Figure 14. Gene set enrichment analysis of significantly heteroskedastic genes (FDR<0.05) ranked by tissue-averaged heteroskedasticity effect size using the MSigDB hallmark pathway gene sets

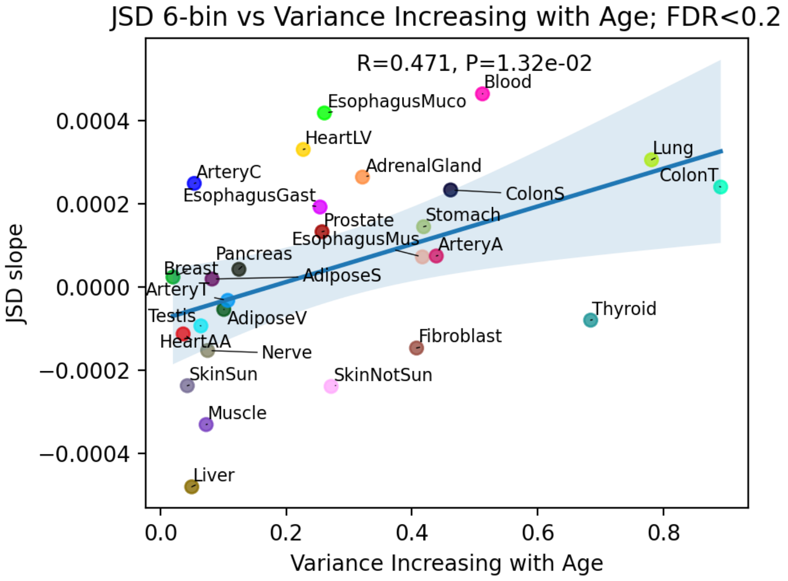

Supplementary Figure 15. JSD age-related heterogeneity metric with 6 age bins vs proportion of heteroskedastic genes with increasing expression variance with age. Error band show bootstrapped 95% confidence interval of slope. Significance determined with two-sided Pearson correlation test

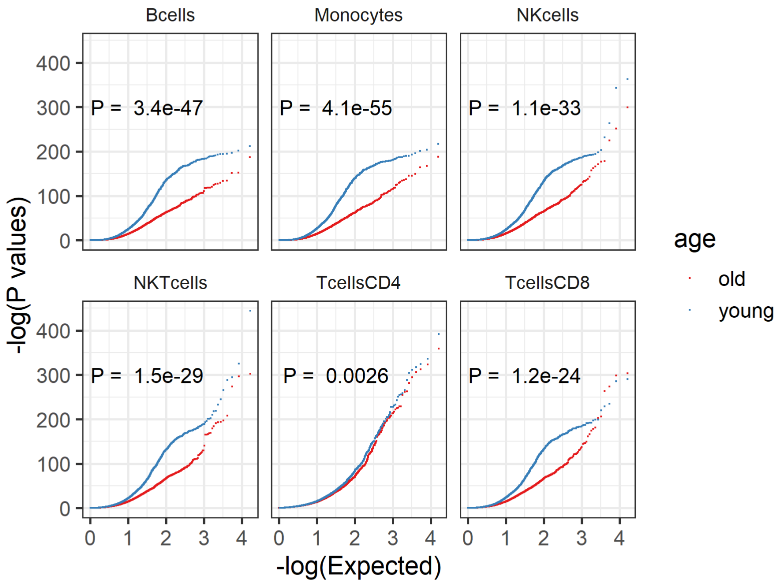

Supplementary Figure 16. QQ plot of eQTL p-values from cell type imputed gene expression profiles of GTEx Whole Blood. Cell type expression levels are estimated from CIBERSORTx high resolution mode using all genes. P-values for distribution difference are obtained from two-sided Welch's t test.

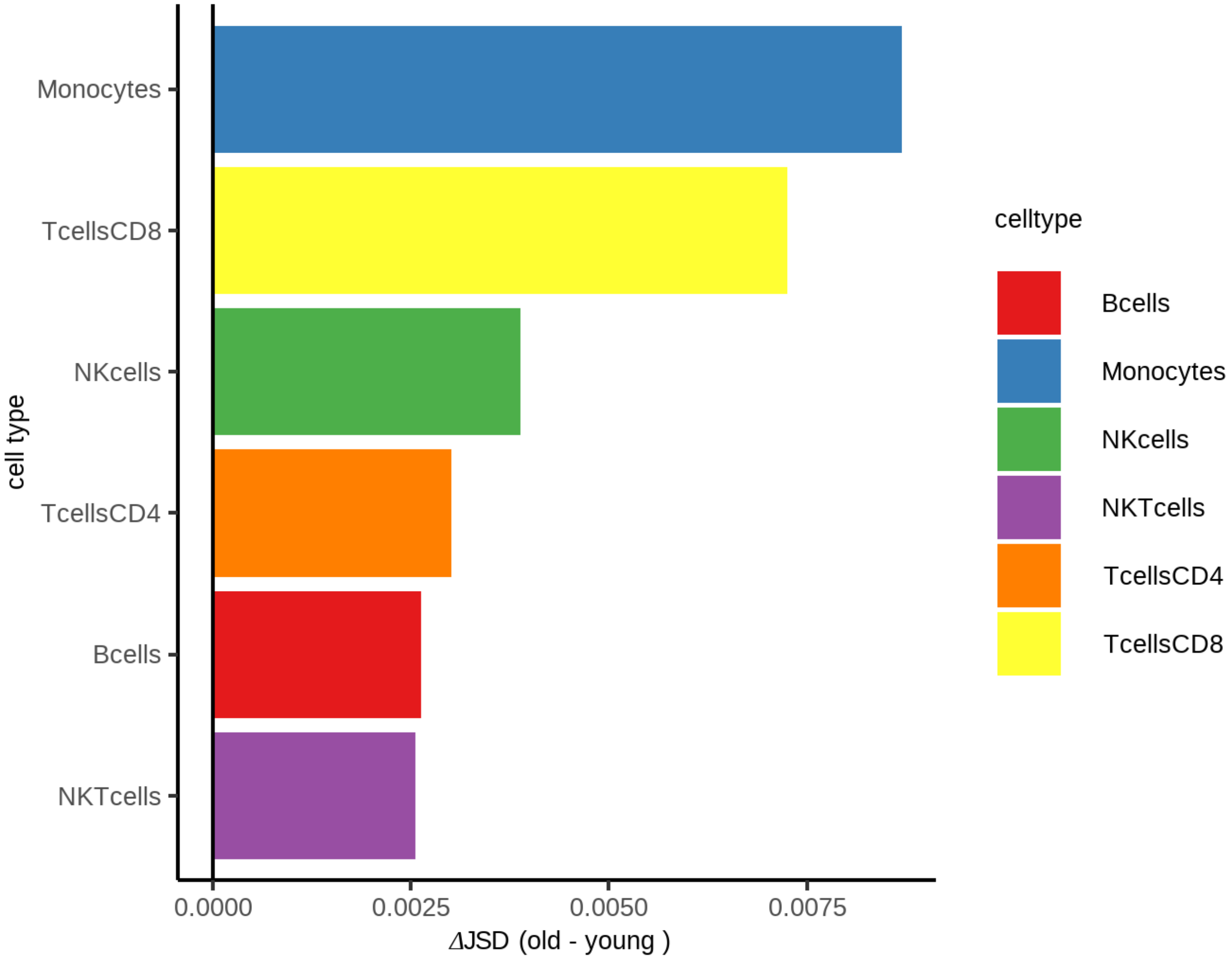

Supplementary Figure 17. JSD applied to individual level cell expression estimates from GTEx Whole Blood with 2 bins. Individual level cell expressions are estimated from CIBERSORTx high resolution mode using all genes.

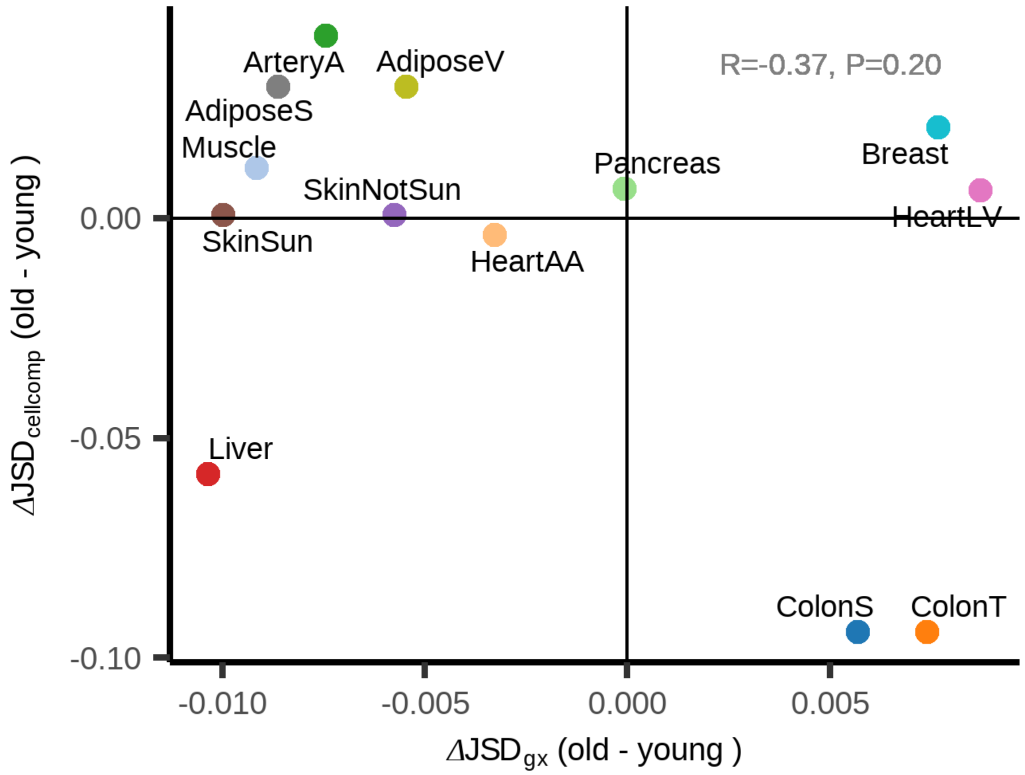

Supplementary Figure 18. JSD heterogeneity metric on transcriptome profile with 2 age bins vs JSD applied to cell proportions. Cell proportion estimates are calculated using CIBERSORT. R and p-value are obtained from two-sided Pearson correlation test.

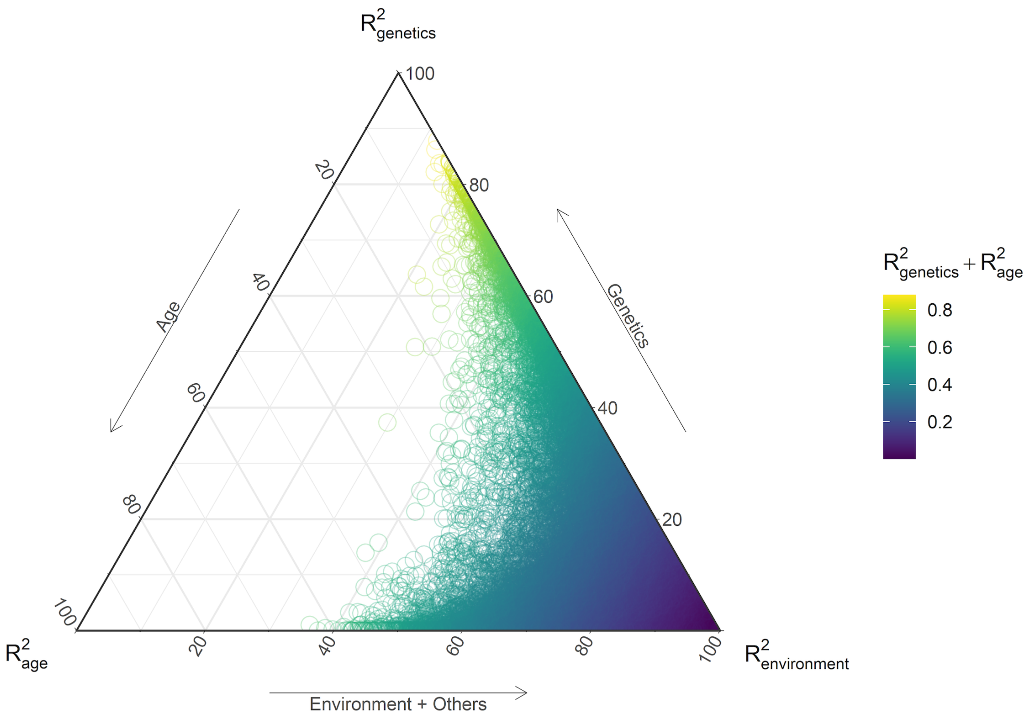

Supplementary Figure 19. Ternary plot of the proportion of each genes expression variance explained by age , genetics and the environment.

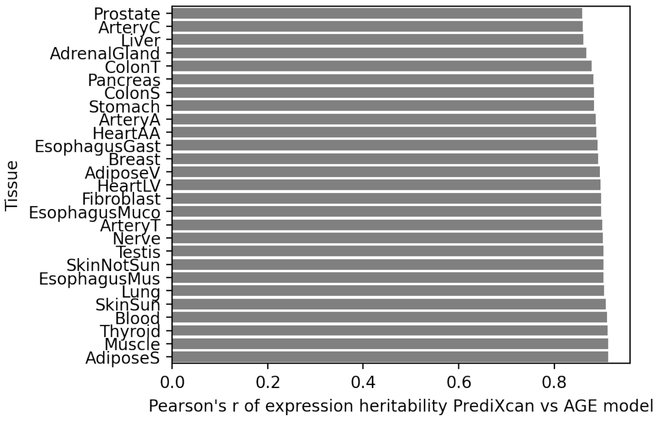

Supplementary Figure 20. Pearson's r of heritability estimate from PrediXcan (PredictDB) vs our model for each tissue

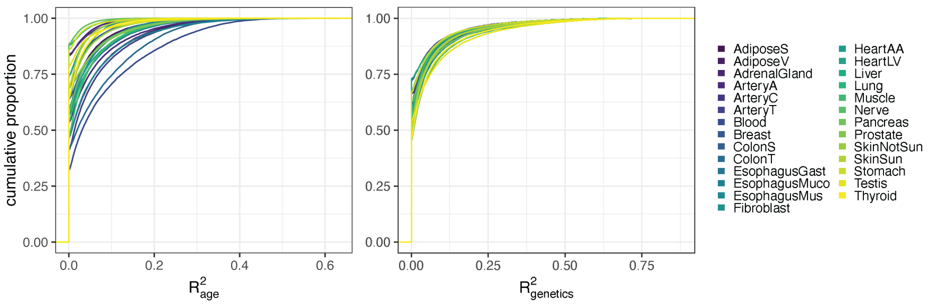

Supplementary Figure 21. Cumulative distribution of R^2_{age} and h^2 for all modeled genes within 27 tissues.

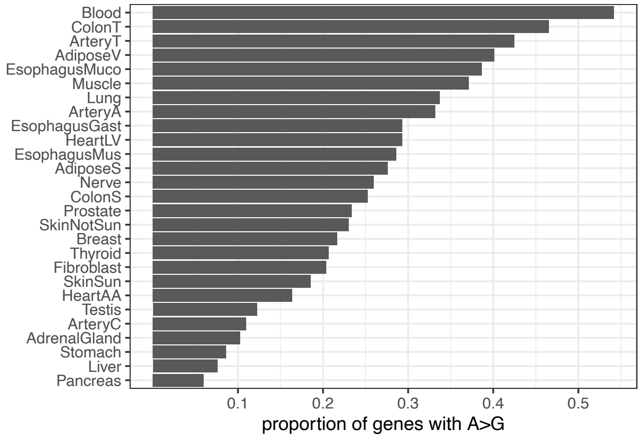

Supplementary Figure 22. Proportion of genes within a tissue that have $R^2_{\text{age}} > h^2$

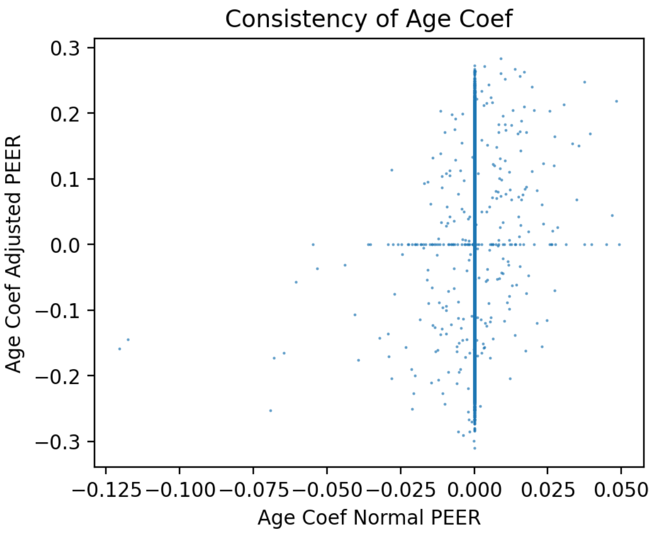

Supplementary Figure 23. Scatter plot of each gene's β_{age} of multiSNP model using GTEx PEER factors vs age-independent PEER factors for Whole Blood.

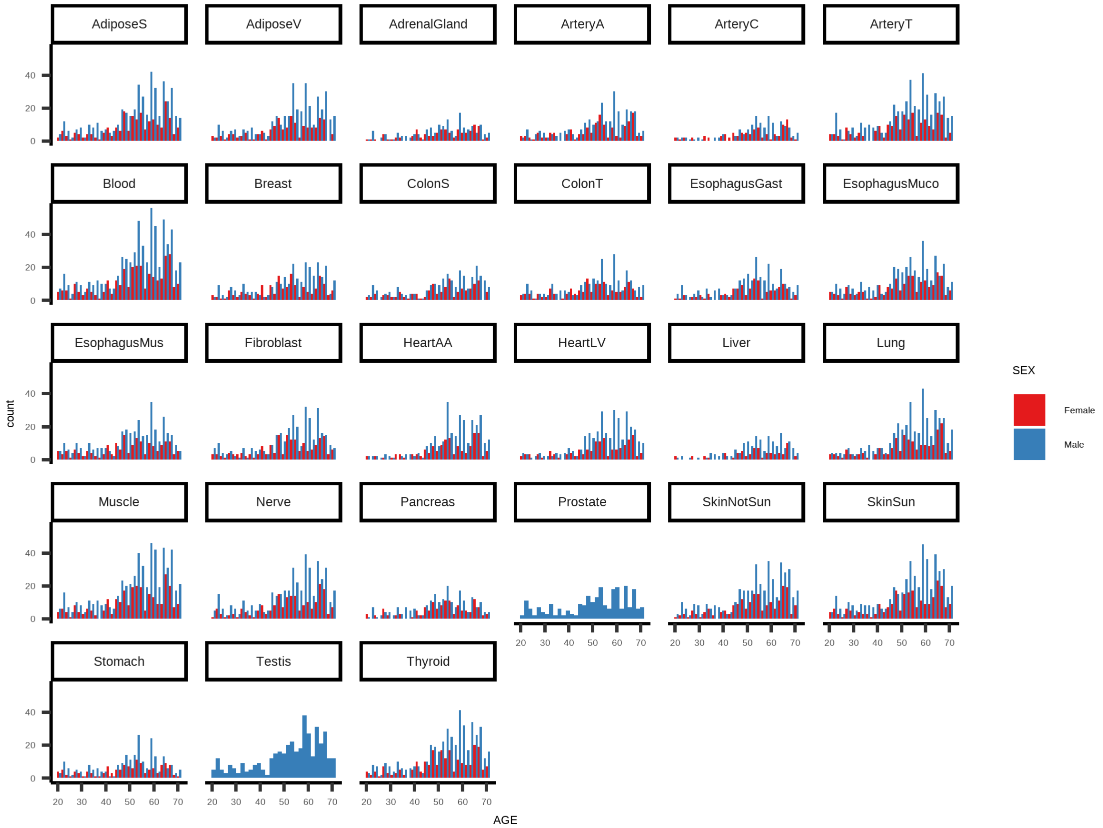

Supplementary Figure 24. Age distribution in each tissue stratified by sex

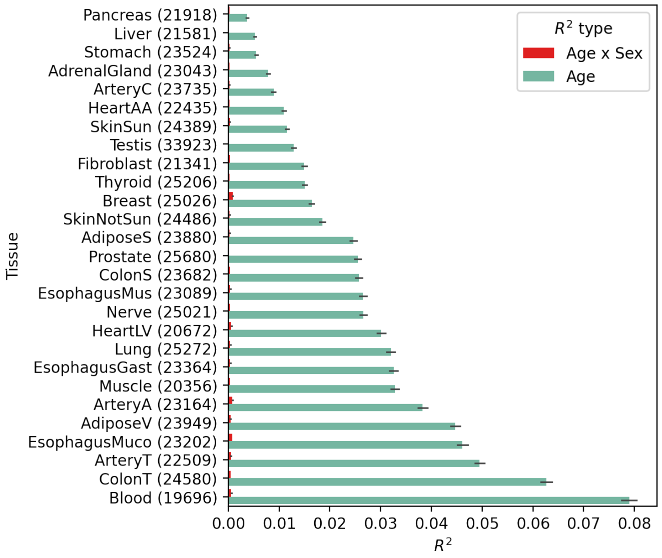

Supplementary Figure 25. Average variance explained (R^2) across all tissues for age term and age-sex interaction term. Error bars indicate standard error of mean. Number of independent genes indicated on y-axis

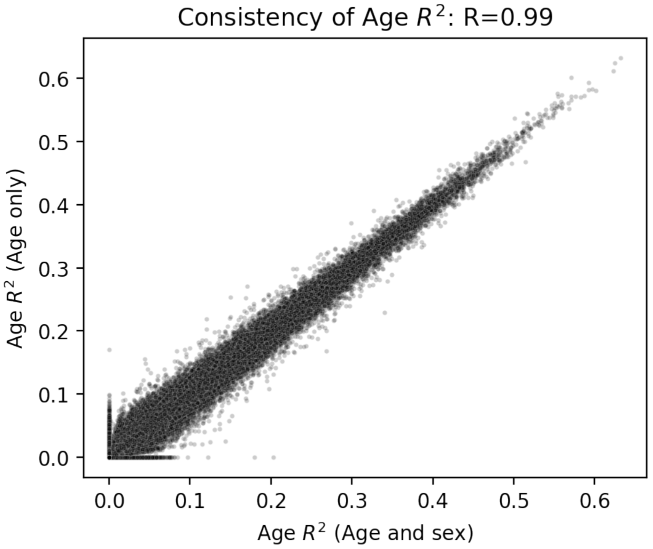

Supplementary Figure 26. Variance explained (R^2) across all genes and tissues for age term for joint age and genetics model and model that includes age-sex interaction term.

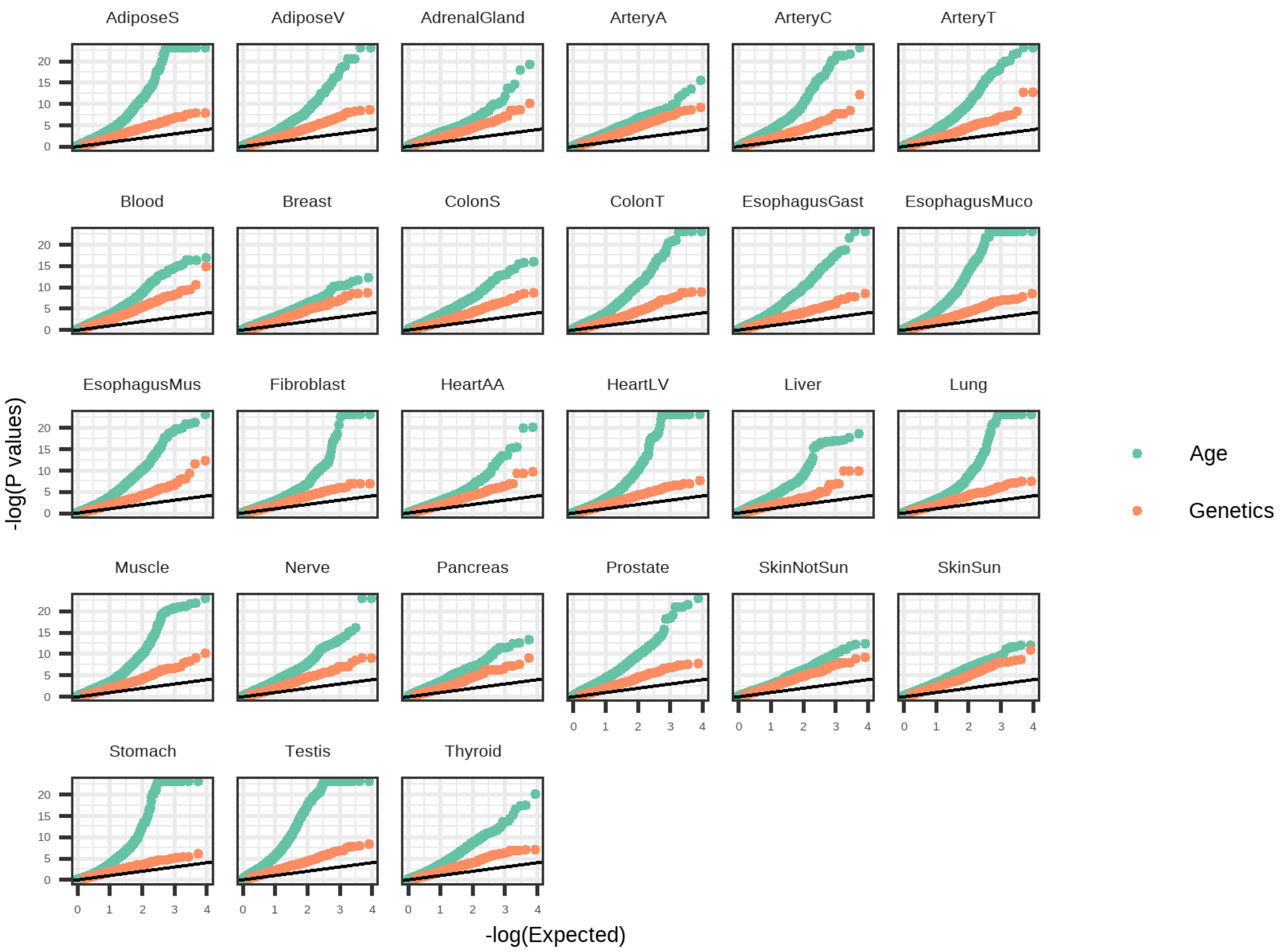

Supplementary Figure 27. GO gene set enrichment P-values across all tissue for genes ranked by either h^2 or R^2_{age}

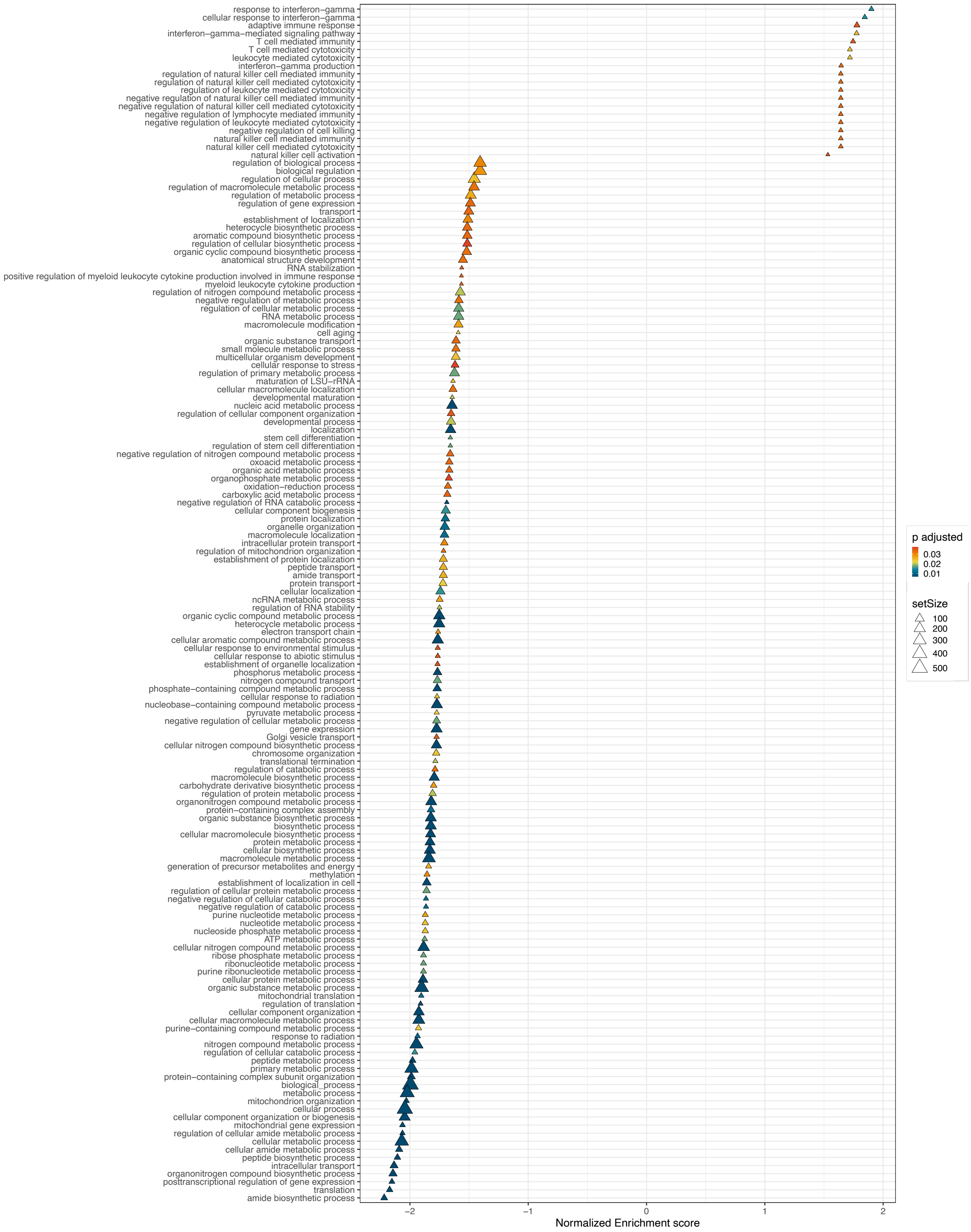

Supplementary Figure 28. Pruned list of 50 GO Biological Processes identified in a GSEA using genes ranked by tissue-average β_{age} (FDR 0.05) (Full set of pathways in Supplementary Data 5)

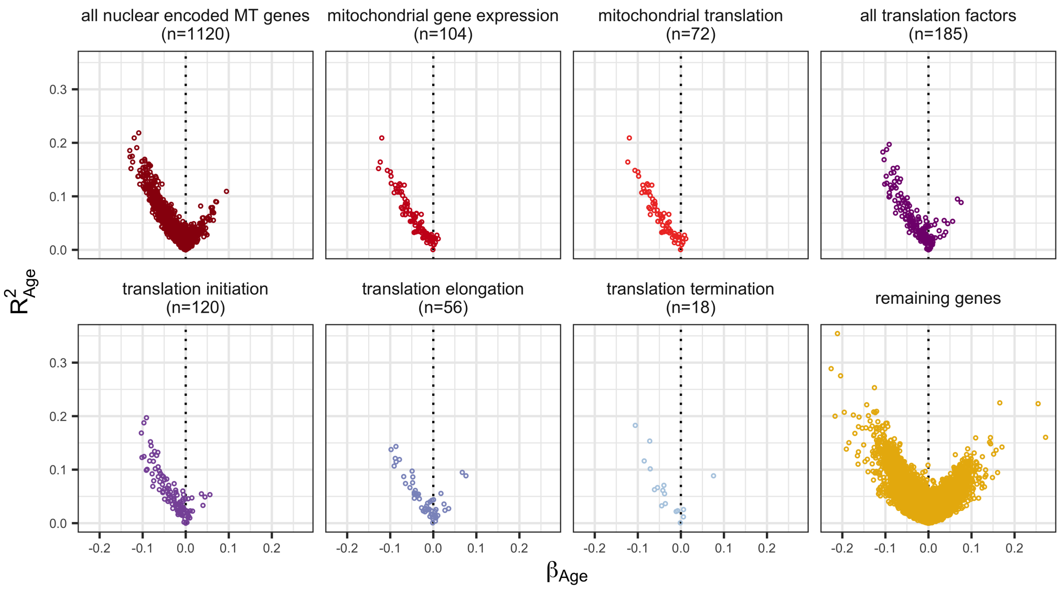

Supplementary Figure 29. Distribution of tissue-averaged β_{age} and R^2 for genes associated with specific mitochondrial and translation pathways

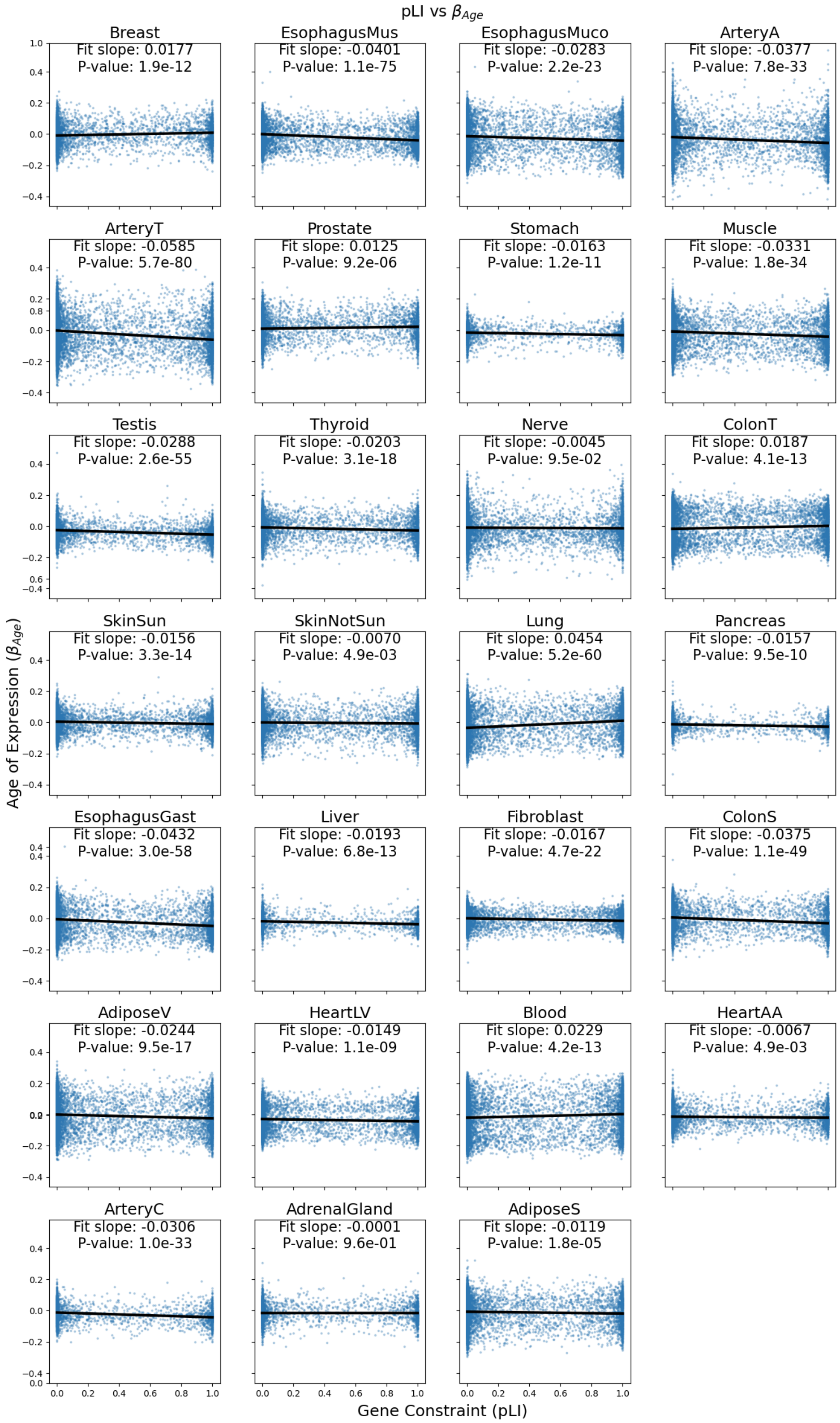

Supplementary Figure 30. Age of expression (β_{Age}) vs gene constraint (pLI) for all tissues with slope of linear fit and p-value of slope. Significance determined using linear model two-sided t-test

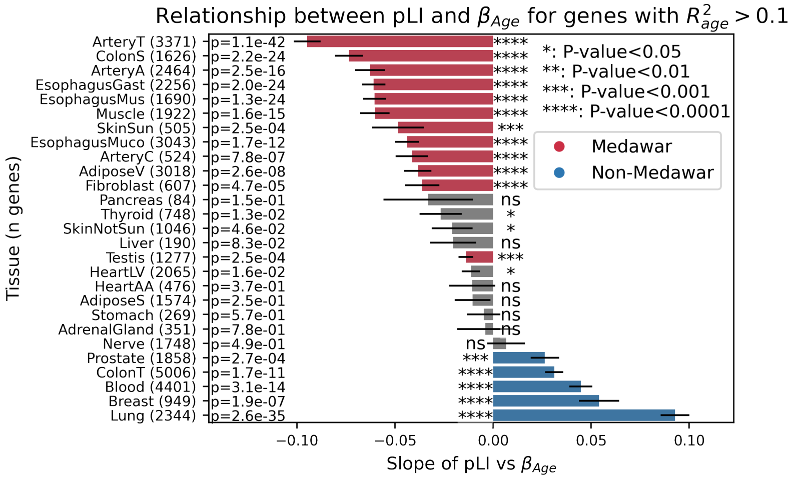

Supplementary Figure 31. The slope of the relationship between gene constraint (pLI) and age of expression (β_{Age}) across tissues for genes with $R^2_{Age} > 0.1$. We repeated the analysis from figure 5D using only genes with $\geq 10\%$ age-related gene expression variance. Color in this figure only indicates consistency with Medawar's hypothesis with this subset of genes (Medawar - negative slope and P<0.001). Significance determined using linear model two-sided t-test. Error bars indicate standard error of the mean. Number of samples and exact pvalues indicated to left of tissue names.

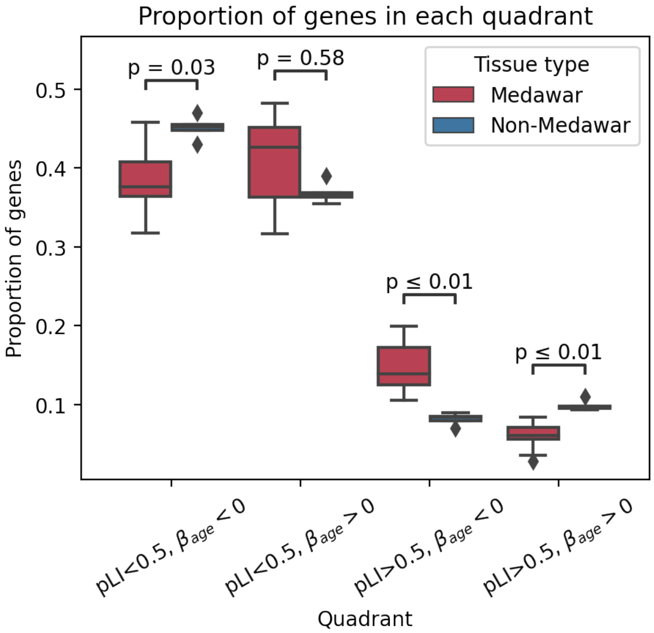

Supplementary Figure 32. More highly constrained, late expressed genes in non-Medawar tissues than in Medawar tissues. We plot the proportion of genes within each quadrant of the gene constraint (pLI) vs. age of expression (β_{age}) plots stratified by whether the tissue showed a significant Medawar or Non-Medawar trend. Significance determined using two-sided Mann-Whitney-Wilcoxon test. Bonferonni corrected p-values shown. Center line of the boxplot indicates median, box limit indicates first and third quartiles, whiskers indicate the maximum and minimum and points indicate outliers. N=24 tissues; 20 Medawar, 4 Non-Medawar.

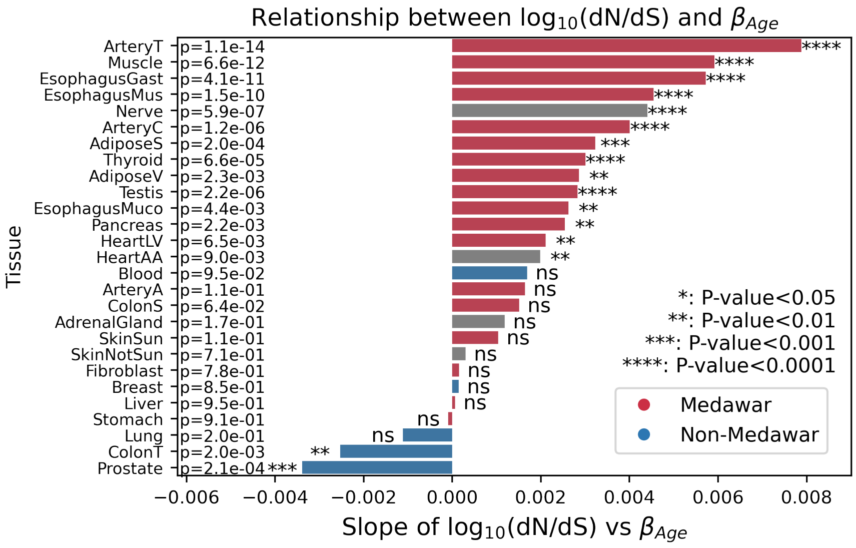

Supplementary Figure 33. Linear regression slope of evolutionary constraint (dN/dS data from ortholog comparison between 8,175 human and chimpanzee genes) vs age-associated gene expression (β_{age}) across tissues. Genes subsetted to $R^2_{\mathrm{age}} > 0$. Significance determined using linear model two-sided t-test. Exact p-values provided to right of tissue names.

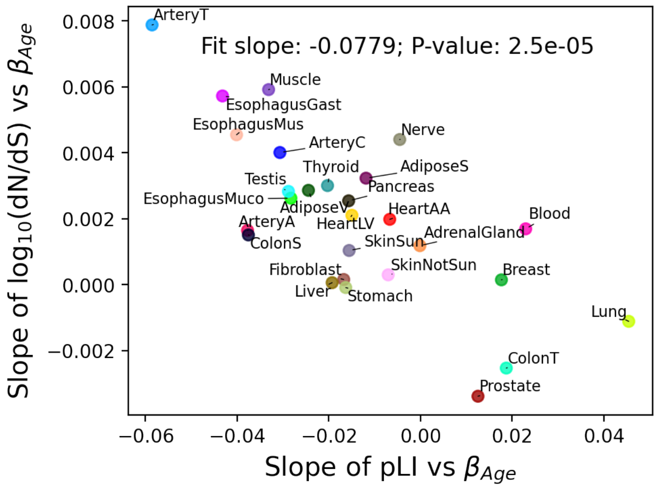

Supplementary Figure 34. Consistency of Medawarian trend measures. We plot the slope of gene constraint metrics (pLI and dN/dS) vs age of expression (β_{age}) for each tissue. Significance determined using linear model two-sided t-test.

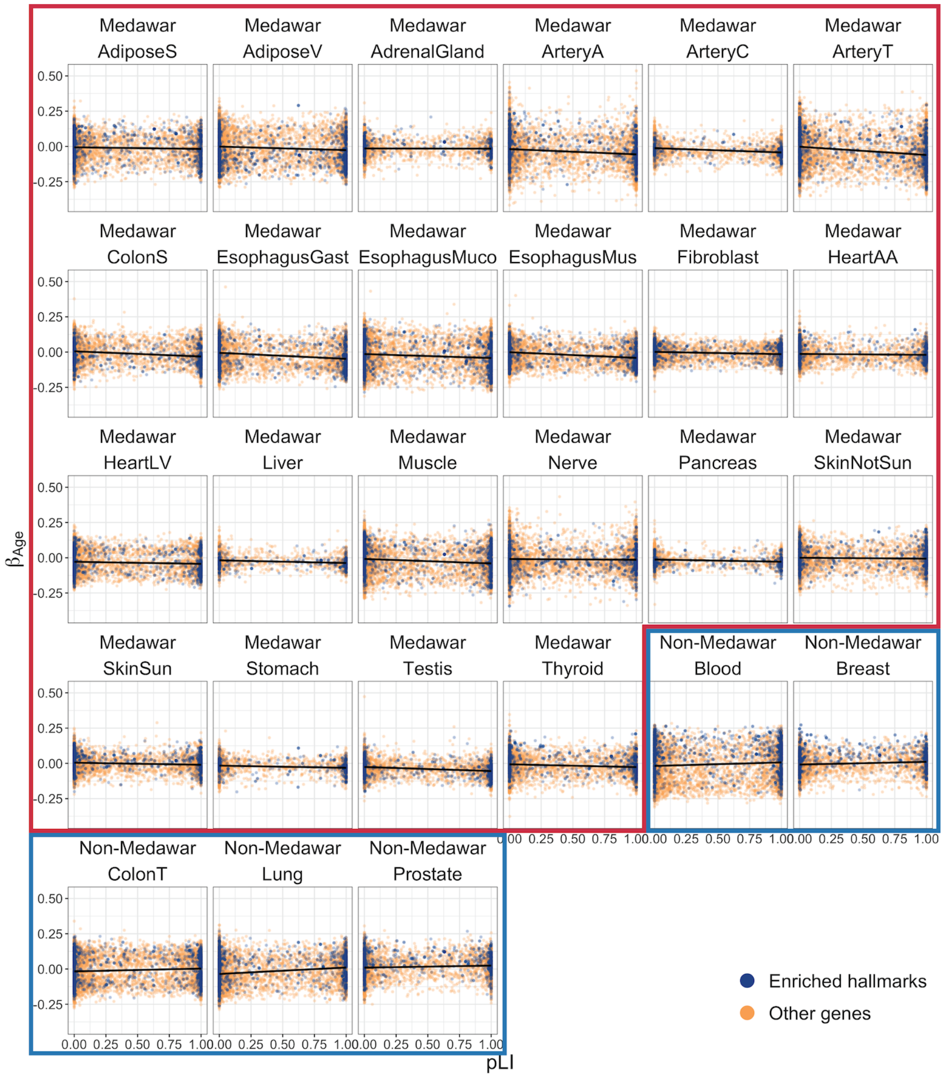

Supplementary Figure 35. The relationship between β_{age} and pLI among tissues showing the strongest *Medawarian* and *non-Medawarian* signatures with genes in halmark pathways from (**5D**) highlighted in blue.

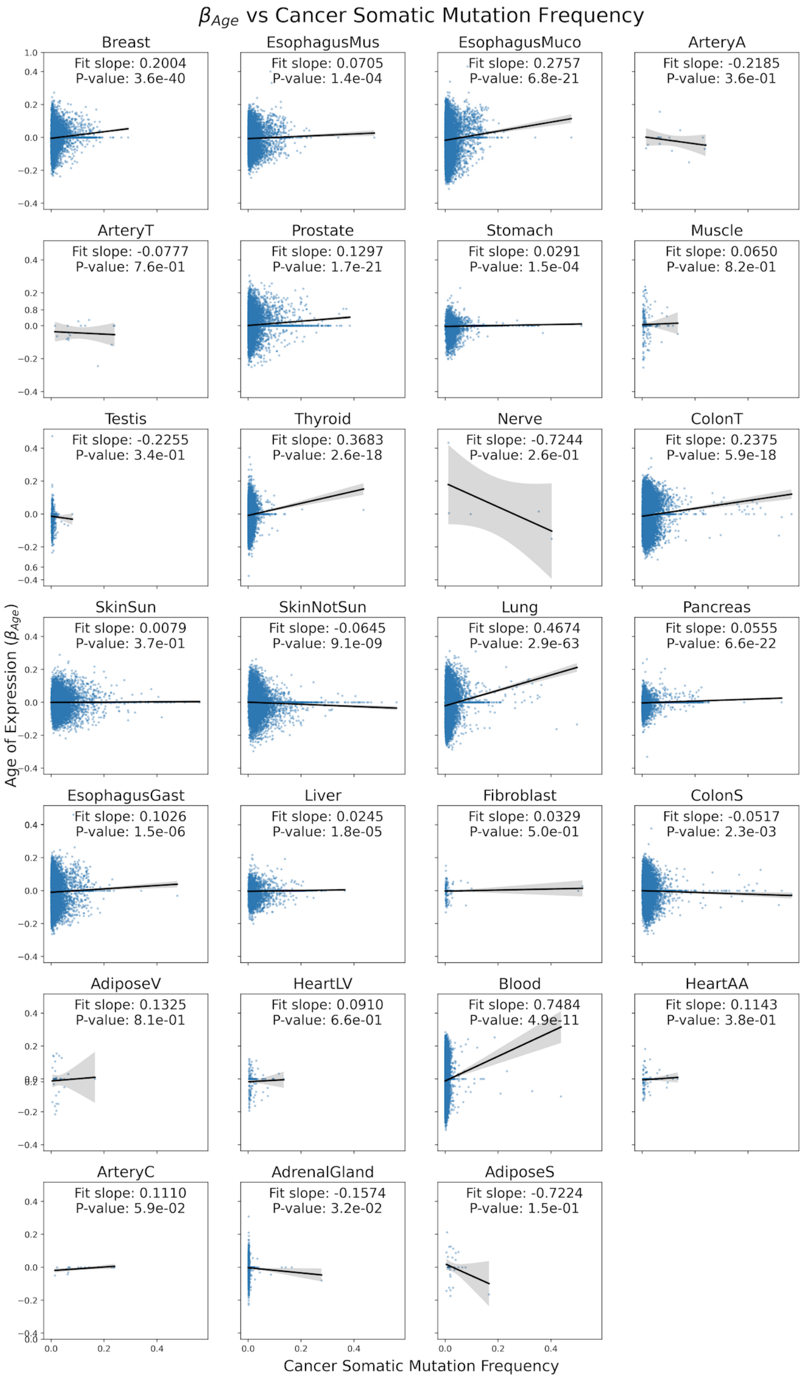

Supplementary Figure 36. Age of expression (β_{Age}) vs proportion of tumor samples with somatic mutation in gene across all tissues with slope of linear fit and p-value of slope. Genes included if they contain at least one sample with a somatic mutation within that gene. Error bands show bootstrapped 95% confidence interval of slope. Significance determined using linear model two-sided t-test.

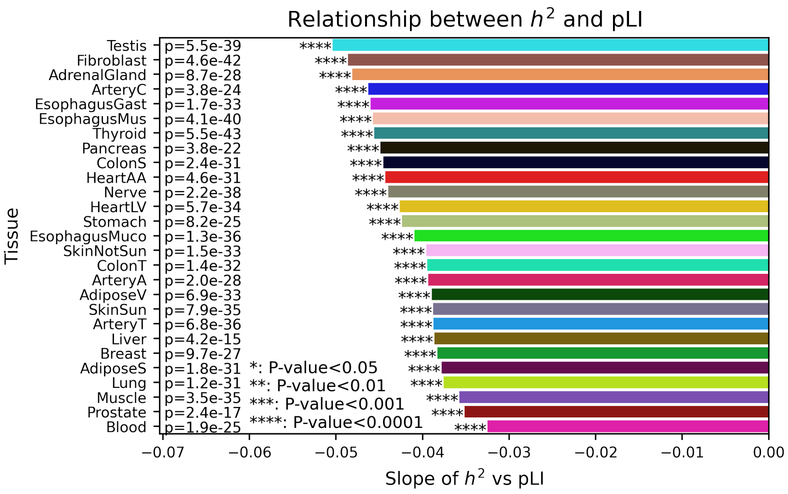

Supplementary Figure 37. The slope of the relationship between constraint (pLI) and heritability (h^2) across tissues. Slope and significance calculated from distribution of heritability and constraint for all tested genes (Supplementary Figure 38). Significance determined using linear model two-sided t-test.

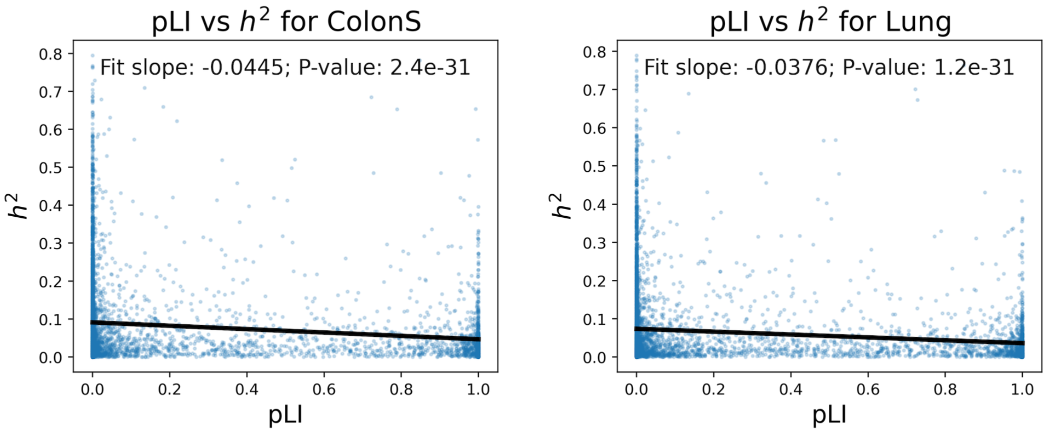

Supplementary Figure 38. Distribution of gene expression heritability vs gene constraint (pLI) for all analyzed genes within two tissues. Slope and significance of relationship indicated. Significance determined using linear model two-sided t-test.

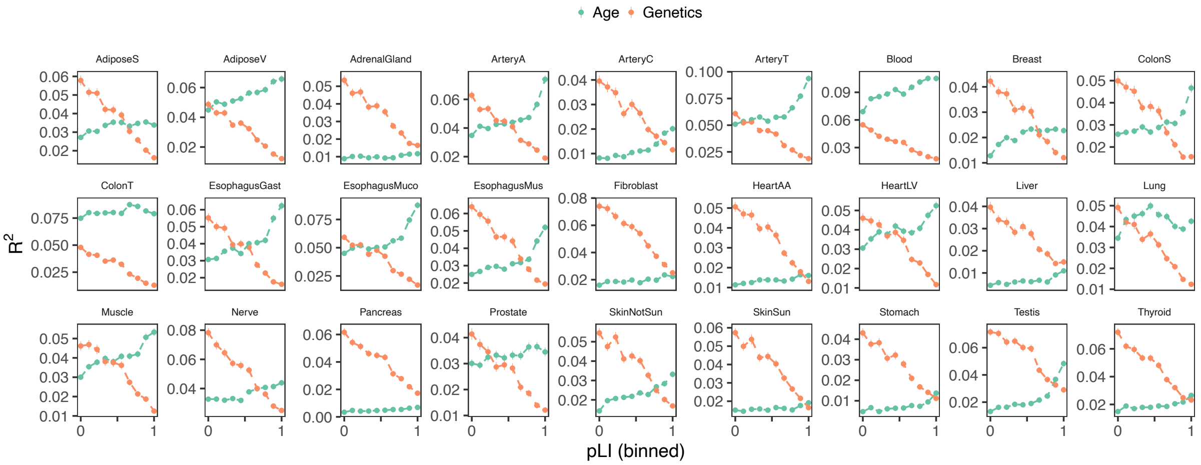

Supplementary Figure 39. Relationship between gene expression variance explained by age and genetics binned by gene constraint (pLI) across tissues. Error bars indicate standard error of the mean. Number of genes analyzed per tissue indicated in figure 5c

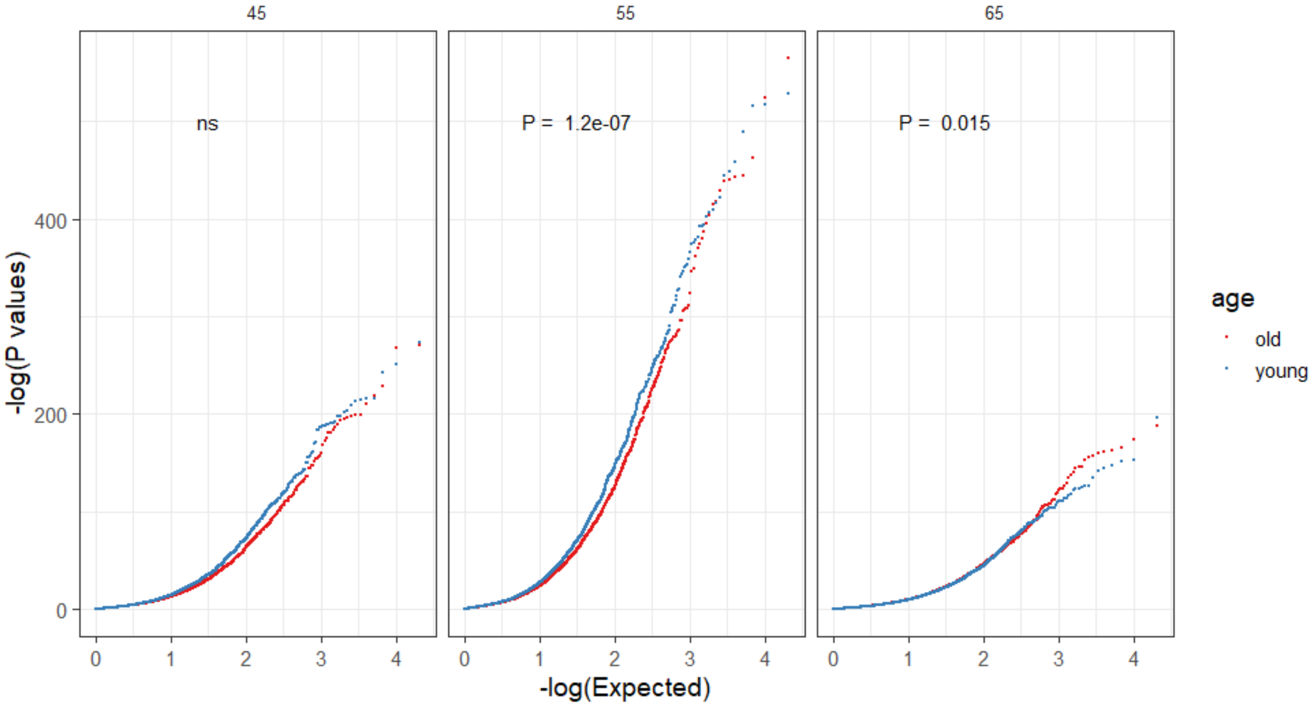

Supplementary Figure 40. QQ plot of eQTL p-values from GTEx Whole Blood using different age cutoffs for two age bins, 45, 55 and 65 years old respectively. P-values for distribution difference are obtained from two-sided Welch's t test.

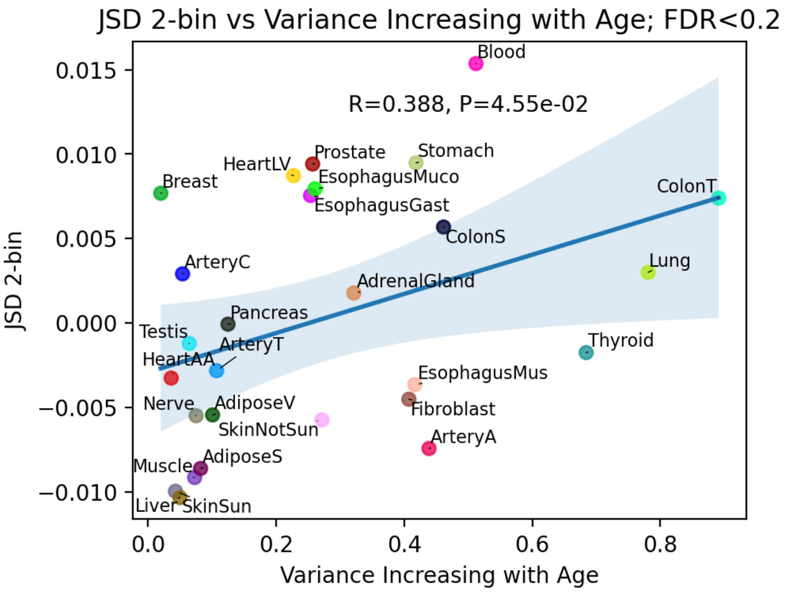

Supplementary Figure 41. JSD age-related heterogeneity metric with 2 age bins vs proportion of heteroskedastic genes with increasing expression variance with age. Error band show bootstrapped 95% confidence interval of slope. Significance determined using linear model two-sided t-test.

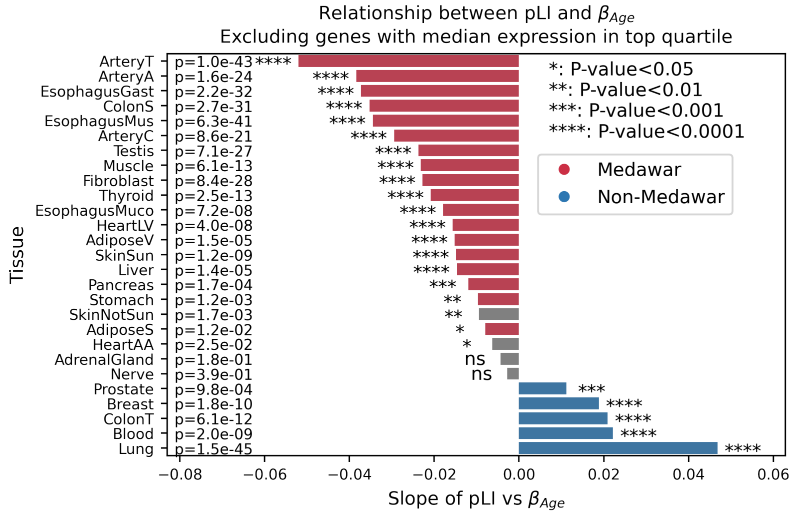

Supplementary Figure 42. The slope of the relationship between gene constraint (pLI) and age of expression (β_{age}) across tissues excluding highly expressed genes. We repeated the analysis from figure 5D using genes from the lower 3 quartiles of median gene expression from GTEx. Color indicates consistency with Medawar's hypothesis from figure 5D. Spearman's ρ 0.98 between slopes in 5D and this figure. Significance determined using linear model two-sided t-test.

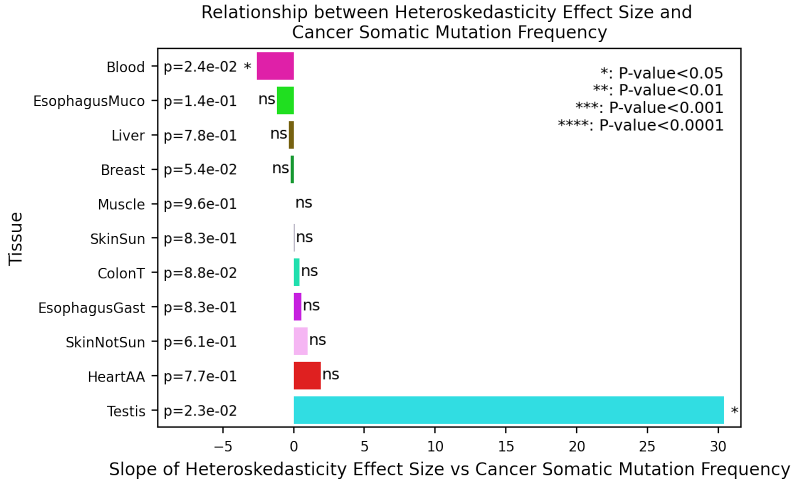

Supplementary Figure 43. Effect size of age-related heteroskedasticity vs cancer somatic mutation frequency. Positive relationship indicates that genes with higher expression variance in old have higher rate of cancer somatic mutations. Only genes with >200 sequenced tumor samples in COSMIC and significant heteroskedasticity (FDR<0.2) are used. Tissues with $\leq$5 genes meeting criterion are not show. Significance determined using linear model two-sided t-test.

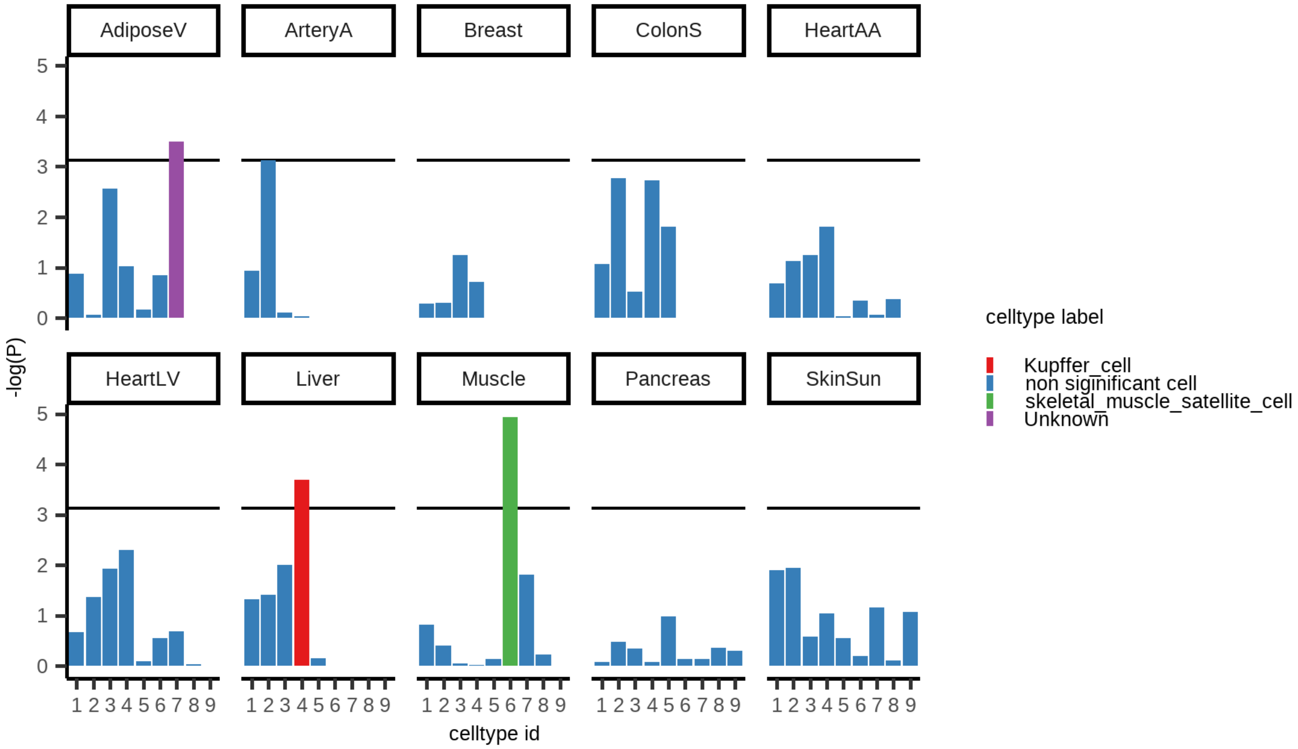

Supplementary Figure 44. Breusch-Pagan heteroskedasticity test P-value for each cell proportion on each tissue. Horizontal line represents Bonferroni cutoff and only significant cell types are labeled.
