# Supplementary material for: Tissue-specific impacts of aging and genetics on gene expression patterns in humans
Source: Nat Commun. 2022 Oct 3;13:5803. doi: 10.1038/s41467-022-33509-0 (PMC9530233; doi:10.1038/s41467-022-33509-0)
Supplement: Supplementary file 2 — Description of Additional Supplementary Files [file 41467_2022_33509_MOESM2_ESM.pdf]

Description of Additional Supplementary Files

File Name: Supplementary Data 1
Description: Number of individuals in "old" and "young" cohorts

File Name: Supplementary Data 2
Description: Names of GTEx tissues with matching abbreviations used within figures

File Name: Supplementary Data 3
Description: Genes with significant age-related gene expression heteroscedasticity

File Name: Supplementary Data 4
Description: Genes with nonzero age-sex interaction term

File Name: Supplementary Data 5
Description: GO enrichment of R^2 of age across all tissues

File Name: Supplementary Data 6
Description: Tissues with matching COSMIC query and number of sequenced tumor samples
.
