# Supplementary material for: Tissue-specific impacts of aging and genetics on gene expression patterns in humans
Source: Nat Commun. 2022 Oct 3;13:5803. doi: 10.1038/s41467-022-33509-0 (PMC9530233; doi:10.1038/s41467-022-33509-0)
Supplement: Supplementary file 4 — Reporting Summary [file 41467_2022_33509_MOESM4_ESM.pdf]

nature portfolio

Corresponding author(s): Nilah Ioannidis, Peter Sudmant

Last updated by author(s): May 10, 2022

Reporting Summary

Nature Portfolio wishes to improve the reproducibility of the work that we publish. This form provides structure for consistency and transparency in reporting. For further information on Nature Portfolio policies, see our Editorial Policies and the Editorial Policy Checklist.

Statistics

For all statistical analyses, confirm that the following items are present in the figure legend, table legend, main text, or Methods section.

n/a	Confirmed	
☐	☒	The exact sample size (*n*) for each experimental group/condition, given as a discrete number and unit of measurement
☐	☒	A statement on whether measurements were taken from distinct samples or whether the same sample was measured repeatedly
☐	☒	The statistical test(s) used AND whether they are one- or two-sided<br>*Only common tests should be described solely by name; describe more complex techniques in the Methods section.*
☐	☒	A description of all covariates tested
☐	☒	A description of any assumptions or corrections, such as tests of normality and adjustment for multiple comparisons
☐	☒	A full description of the statistical parameters including central tendency (e.g. means) or other basic estimates (e.g. regression coefficient) AND variation (e.g. standard deviation) or associated estimates of uncertainty (e.g. confidence intervals)
☐	☒	For null hypothesis testing, the test statistic (e.g. *F*, *t*, *r*) with confidence intervals, effect sizes, degrees of freedom and *P* value noted<br>*Give P values as exact values whenever suitable.*
☒	☐	For Bayesian analysis, information on the choice of priors and Markov chain Monte Carlo settings
☐	☒	For hierarchical and complex designs, identification of the appropriate level for tests and full reporting of outcomes
☐	☒	Estimates of effect sizes (e.g. Cohen's *d*, Pearson's *r*), indicating how they were calculated

Our web collection on statistics for biologists contains articles on many of the points above.

Software and code

Policy information about availability of computer code

Data collection	N/A
Data analysis	Data analyzed using the following existing programs and packages R 4.0.2, PEER 1.3 (R package), Python 3.6.13, Numpy 1.19.5, Scikitlearn 0.24.2, Pandas 1.1.5, glmnet 2.2.1, gcta 1.94.1, CIBERSORTx(https://cibersortx.stanford.edu/) . Custom code for our analysis is provided at https://github.com/sudmantlabgene_expression_aging and archived at https://doi.org/10.5281/zenodo.6555500.

For manuscripts utilizing custom algorithms or software that are central to the research but not yet described in published literature, software must be made available to editors and reviewers. We strongly encourage code deposition in a community repository (e.g. GitHub). See the Nature Portfolio guidelines for submitting code & software for further information.

Data

Policy information about availability of data

All manuscripts must include a data availability statement. This statement should provide the following information, where applicable:

- Accession codes, unique identifiers, or web links for publicly available datasets
- A description of any restrictions on data availability
- For clinical datasets or third party data, please ensure that the statement adheres to our policy

Gene expression measurements and covariates are publically available and were obtained from GTEx V8 using https://gtexportal.org/. Individual genetic and age data is protected and available through AnVIL with a dbGaP application. Gene expression measurements and genotype for PIVUS cohort are available in European Genome Archive under EGAD00001004965.

Field-specific reporting

Please select the one below that is the best fit for your research. If you are not sure, read the appropriate sections before making your selection.

☒ Life sciences ☐ Behavioural & social sciences ☐ Ecological, evolutionary & environmental sciences

For a reference copy of the document with all sections, see nature.com/documents/nr-reporting-summary-flat.pdf

Life sciences study design

All studies must disclose on these points even when the disclosure is negative.

Sample size	We used all available samples for each tissue included in analysis for GTEx and PIVUS cohort.
Data exclusions	Of the 54 tissues within GTEx Analysis Release V8 we excluded 27 tissues with a low number of individuals (<100 samples with genotype and expression data) within either the young or old cohorts. The threshold of 100 samples was used to provide enough statistical power to call eQTL associations. The sample cutoff is more conservative than the GTEx V8 sample cutoff of 70 samples.
Replication	We replicated results from our analyses of GTEx blood gene expression using the PIVUS cohort.
Randomization	When the number of individuals within the old and young cohorts did not match, individuals were randomly sampled. Samples were randomly divided into training/validation sets during cross validation of gene expression prediction model.
Blinding	No blinding was used in this observational study however our initial hypothesis and models made no assumptions about sample status.

Reporting for specific materials, systems and methods

We require information from authors about some types of materials, experimental systems and methods used in many studies. Here, indicate whether each material, system or method listed is relevant to your study. If you are not sure if a list item applies to your research, read the appropriate section before selecting a response.

Materials & experimental systems

n/a	Involved in the study	
☒	☐	Antibodies
☒	☐	Eukaryotic cell lines
☒	☐	Palaeontology and archaeology
☒	☐	Animals and other organisms
☒	☐	Human research participants
☒	☐	Clinical data
☒	☐	Dual use research of concern

Methods

n/a	Involved in the study	
☒	☐	ChIP-seq
☒	☐	Flow cytometry
☒	☐	MRI-based neuroimaging
